# Poacic Acid, a Plant-Derived Stilbenoid, Augments Cell Wall Chitin Production, but Its Antifungal Activity Is Hindered by This Polysaccharide and by Fungal Essential Metals

**DOI:** 10.1021/acs.biochem.3c00595

**Published:** 2024-03-27

**Authors:** Adi Yona, Micha Fridman

**Affiliations:** School of Chemistry, Raymond & Beverly Sackler Faculty of Exact Sciences, Tel Aviv University, Tel Aviv 6997801, Israel

## Abstract

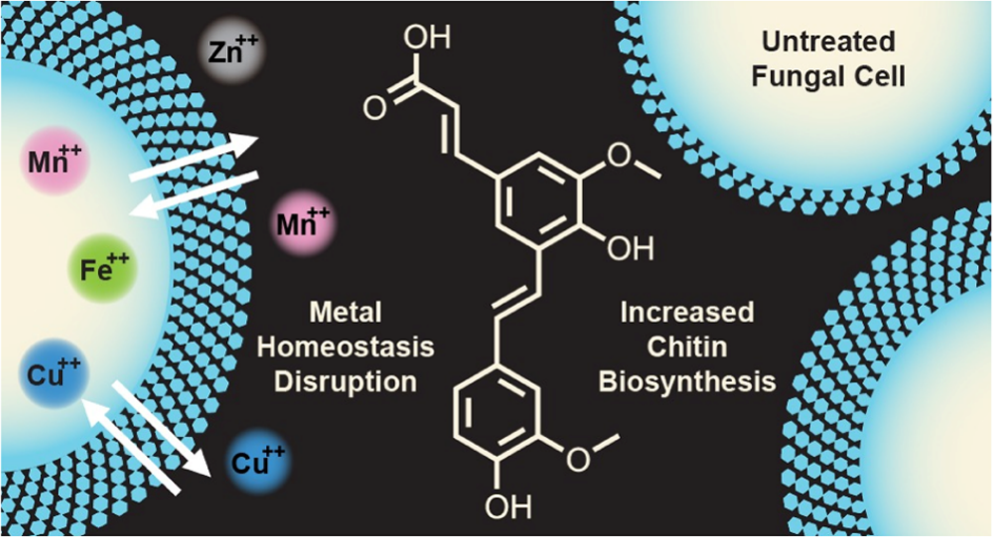

Climate and environmental changes have modified the habitats of fungal pathogens, inflicting devastating effects on livestock and crop production. Additionally, drug-resistant fungi are increasing worldwide, driving the urgent need to identify new molecular scaffolds for the development of antifungal agents for humans, animals, and plants. Poacic acid (PA), a plant-derived stilbenoid, was recently discovered to be a novel molecular scaffold that inhibits the growth of several fungi. Its antifungal activity has been associated with perturbation of the production/assembly of the fungal cell wall β-1,3-glucan, but its mode of action is not resolved. In this study, we investigated the antifungal activity of PA and its derivatives on a panel of yeast. PA had a fungistatic effect on *S. cerevisiae* and a fungicidal effect on plasma membrane-damaged *Candida albicans* mutants. Live cell fluorescence microscopy experiments revealed that PA increases chitin production and modifies its cell wall distribution. Chitin production and cell growth returned to normal after prolonged incubation. The antifungal activity of PA was reduced in the presence of exogenous chitin, suggesting that the potentiation of chitin production is a stress response that helps the yeast cell overcome the effect of this antifungal stilbenoid. Growth inhibition was also reduced by metal ions, indicating that PA affects the metal homeostasis. These findings suggest that PA has a complex antifungal mechanism of action that involves perturbation of the cell wall β-1,3-glucan production/assembly, chitin production, and metal homeostasis.

## Introduction

The increased use of antifungals in both agriculture, for the protection of crops and livestock, and clinic has driven the worldwide emergence and spread of pathogens capable of resisting the current repertoire of antifungal drugs and fungicides.^1^ The global burden of fungal disease poses a substantial threat to human, animal, and environmental health, endangering both human and livestock populations creating vulnerabilities to global food supplies.^2,3^ In humans, infections with pathogenic fungi are a serious health threat with treatment failure ranging from 30 to 90% in Western hospitals. Multidrug-resistant “superbugs” such as *Candida auris* and *Candida glabrata* have emerged and are alarmingly increasing in prevalence worldwide.^4−7^

Fungal pathogens are eukaryotes like their plant or animal hosts, which adds challenges to the development of new side-effects-free and effective antifungal agents.^8,9^ This is reflected by the availability of only three main clinically used antifungal drug classes, azoles, polyenes, and echinocandins, compared to at least 12 antibiotic drug classes that target prokaryotes.^8−10^ Moreover, the limited number of antifungal agents and their cross-use between agriculture and health promote the development of resistance and reduce our defenses against fungal diseases. Antifungal-resistant strains found ubiquitously within the natural environment demonstrate resistance to the same classes of antifungals used to treat human, animal, and plant infections.^2^ This interconnectivity supports the need for a One Health approach to combat fungal diseases and overcome antifungal resistance, ensuring that treatment and protection of humans do not come with the cost of endangering plants and/or animals. To stay ahead in the molecular arms race against pathogenic fungi, the discovery of novel molecular scaffolds with antifungal activity is highly sought after and raises high interest.^2,11−13^

**Scheme 1:**
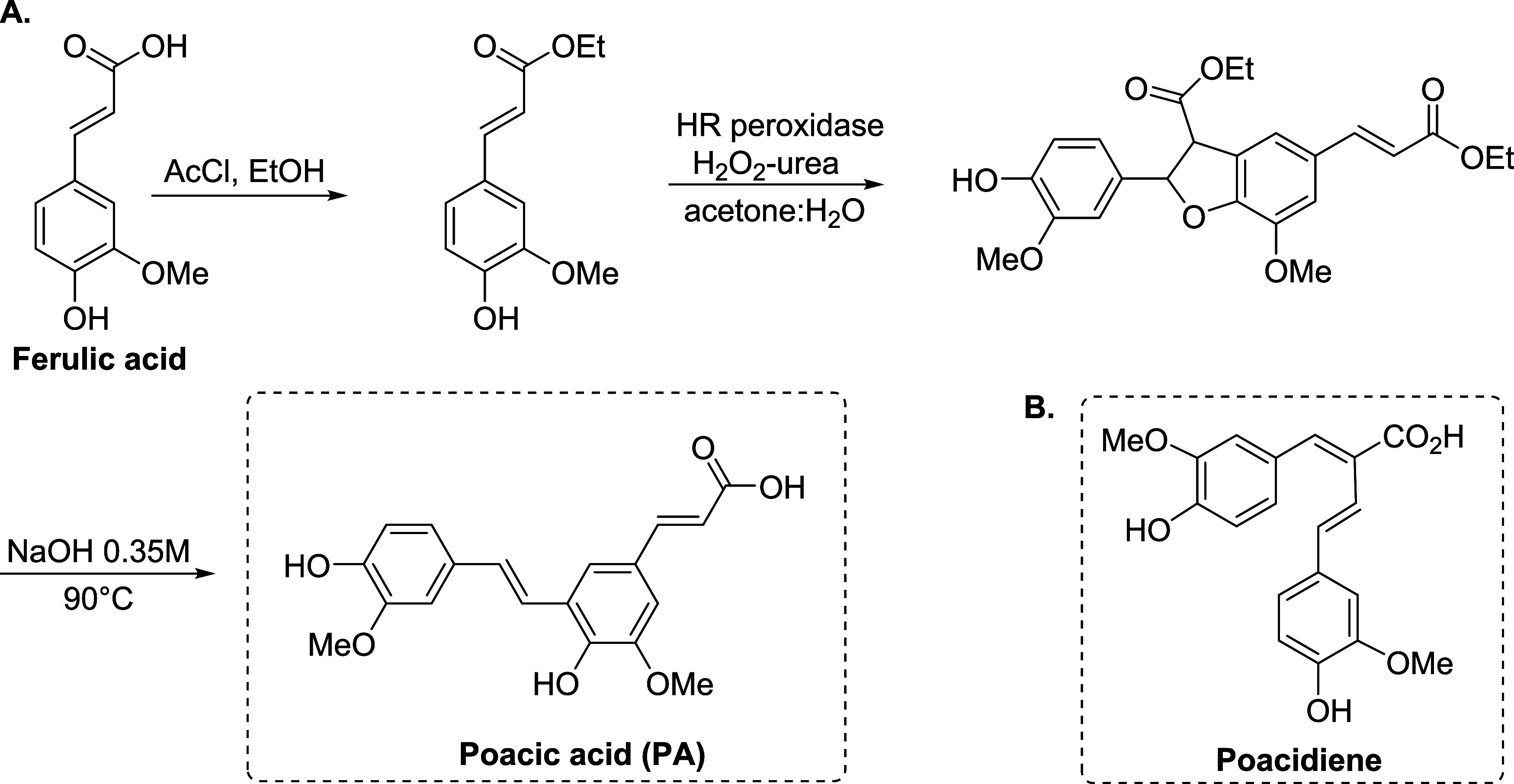
(A) Chemoenzymatic Synthesis of the Plant-Derived *trans*-Stilbenoid Poacic Acid from Ferulic Acid; (B) Structure of the *cis* Stilbenoid Poacidiene

By screening a collection of diferulates found in lignocellulosic hydrolysates for potential antifungal activity against the baker’s yeast *Saccharomyces cerevisiae*, used as a discovery platform, in 2015, Ohya and co-workers identified the plant-derived stilbenoid poacic acid (PA, Scheme 1A) as a novel natural antifungal agent.^14^

More than 400 stilbenoids originating from the plant kingdom have been structurally identified to date, many of which display a variety of biological activities including antifungal properties.^15,16^ The stilbenoid skeleton consists of two aromatic rings attached by an ethylene bridge, thus orienting in either *trans* (*E*) or *cis* (*Z*)-configurations. PA is a decarboxylated product from 8-5-diferulic acid.^14^ Ferulic acid (Scheme 1A) is esterified to grass cell wall polysaccharides, notably to arabinoxylans, and dimerization of such ferulate esters provides a pathway for cross-linking polysaccharide chains.^17^ An efficient and reproducible chemoenzymatic process for large-scale production of PA was reported in 2017 by Ralph and co-workers (Scheme 1A).^18,19^

Ohya and co-workers provided evidence supporting that PA localizes to yeast cell wall and perturbs with its biosynthesis and/or assembly, possibly by interacting directly with β-1,3-glucan, a major and essential constituent of the fungal cell wall. This polysaccharide constitutes between 30 and 80% of the mass of the wall.^14^ Chemical genomics using *Saccharomyces cerevisiae* demonstrated that loss of cell wall biosynthesis and maintenance genes conferred increased sensitivity to PA. Morphological analysis revealed that cells treated with PA behaved similarly to cells treated with other cell wall-targeting antifungal drugs and to mutants with deletions in genes involved in processes related to cell wall biogenesis. PA was shown to synergize with caspofungin, the β-1,3-glucan synthase inhibiting echinocandin antifungal drug, and with fluconazole, the ergosterol biosynthesis inhibiting azole antifungal drug.^14,20,21^

PA, which is inherently fluorescent, was shown to localize to the yeast cell wall and suggested to inhibit cell wall formation by directly binding β-1,3-glucan. By following small changes in the metachromatic interaction between PA and cell wall components, Ohya, Arroyo, and co-workers indicated that the affinity of PA to a cell wall polysaccharide mixture containing a high percentage of β-1,3-glucan was ∼30-fold higher than that for chitin. PA was shown to inhibit the yeast glucan-elongating activity of Gas1 and Gas2, glycosidase/trans glycosidases, a wide group of yeast and fungal enzymes involved in cell wall assembly, and of chitin-glucan transglucosylase activity of Crh1.^21,22^ In response to PA, parallel activation of the cell wall integrity and high-osmolarity glycerol signaling pathways was detected. The transcriptional profiles and regulatory circuits activated by the echinocandin caspofungin, were different than that of PA suggesting that they affect the integrity of the cell wall via different mechanisms.^14,20,21^

In 2022, another diferulate derivative, poacidiene (Scheme 1B), was identified as a novel antifungal agent by Ohya and co-workers.^23^ Surprisingly, despite the high molecular similarity with PA, experimental evidence suggests that their mechanisms of action are fundamentally different. Morphological profiling of yeast cells treated with poacidiene implied that poacidiene impacts DNA damage response and not the yeast cell wall integrity, which was also supported by cell morphology and genetic analysis.^23,24^ To shed light on the biological activities of PA and to examine its value as a cell-wall-directing agent, in this study, we investigated the mode of action of this natural stilbenoid and its synthetic derivatives, revealing new insights on its unique effects on yeast cells.

### PA Exhibits Fungistatic Activity to *S. cerevisiae* and Fungicidal Activity against Membrane Compromised *C. albicans*

The effect of PA on fungal cell growth was evaluated by the broth double-dilution assay on a panel of representative yeast strains including two *S. cerevisiae* strains, and three strains of *Candida* representing different species of this genus of pathogenic yeast.

**Figure 1:**
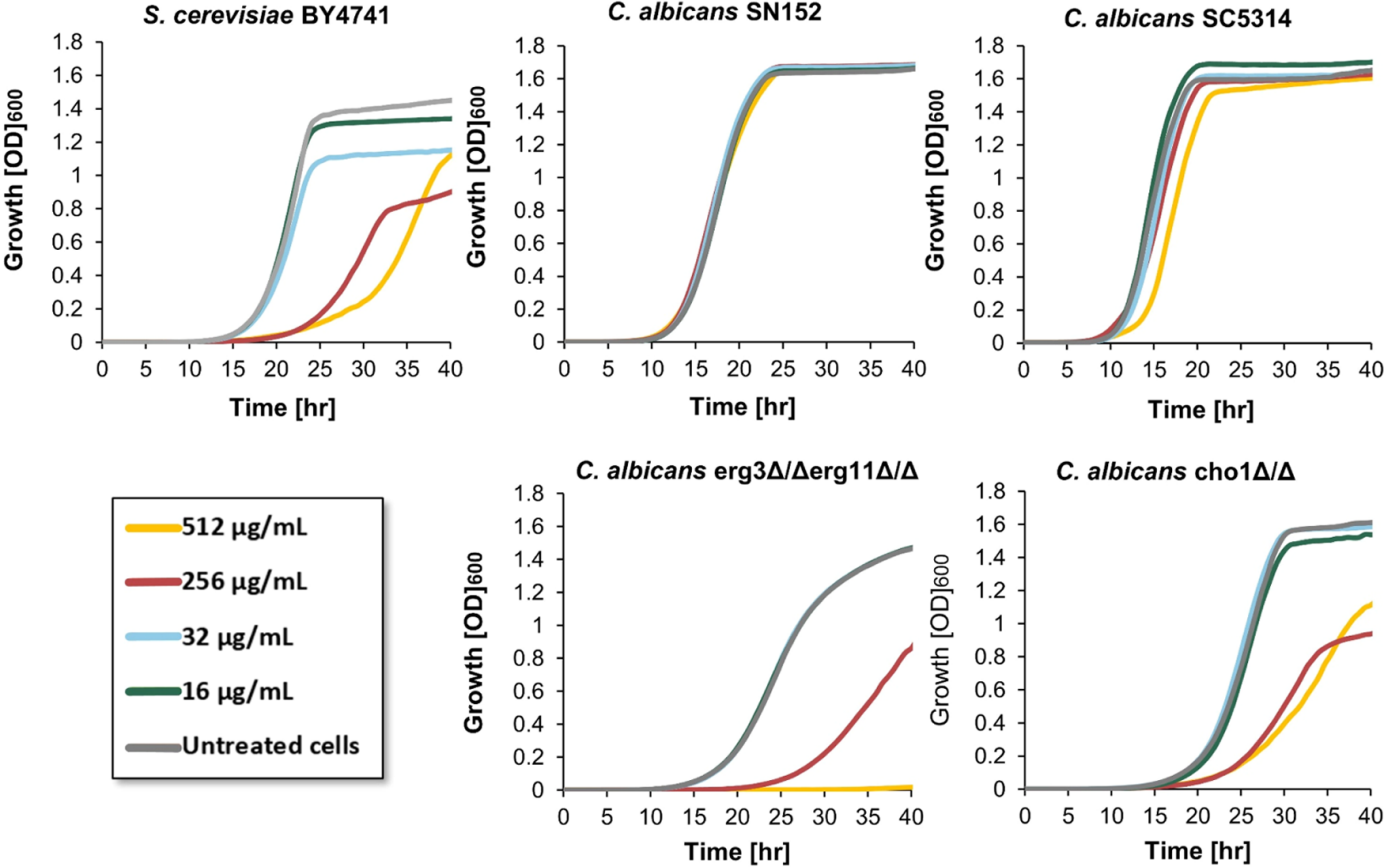
Effects of PA on yeast cell growth. Cells were grown in YPD media at 35 °C and treated with different concentrations of PA. Growth was measured by recording the OD_600_ values on an automated plate reader every 40 min over a 40 h time course. Each experiment was done in triplicate and the results were repeated in two independent experiments.

PA inhibited the growth of the two *S. cerevisiae* strains tested and did not affect the growth of the tested *Candida* strains (Figure 1, Figure S1, and Table S1). Of note, while no significant *S. cerevisiae* yeast cell growth was measured during the first 20 h of incubation with PA at a concentration ≥256 μg/mL, growth resumed, albeit slower compared to untreated cells, after longer incubation. This indicates that the measured inhibition of growth by PA in *S. cerevisiae* is not the result of fungicidal activity, in agreement with observations reported by Gow and co-workers.^20^ The opportunistic human fungal pathogen *C. glabrata* is closely related to *S. cerevisiae*, yet has evolved to survive within mammalian hosts,^25^ and despite their close genetic background, PA significantly inhibited the growth of the latter only. The main differences that grant *C. glabrata* its adaptation to a mammalian cell environment include an extended repertoire of adhesins, high drug resistance, ability to sustain prolonged starvation, and adaptations of their stress response activation pathways.^26^ Likely, one or more of these adaptation mechanisms, which are found in other members of the genus *Candida* as well as in other fungal pathogens, are responsible for PA resistance.

We next asked if alterations in the plasma membrane composition of *C. albicans* strains that were resistant to PA will render them susceptible to this stilbenoid. The growth inhibition effect of PA was tested against *C. albicans* SN152 and its mutant strain lacking copies of the *ERG11* and *ERG3* genes. *ERG11* encodes CYP51, an enzyme in the biosynthesis pathway of ergosterol and the target of antifungal azoles, which is essential for fungal cell growth under aerobic conditions when *ERG3*, which encodes a C-5 sterol desaturase, is functional.^8,27,28^ Therefore, an *erg3ΔΔ/erg11Δ*Δ mutant strain is viable despite the absence of CYP51 yet lacks ergosterol as its plasma membrane sterol, leading to a more permeable cell. This strain exemplifies resistance to antifungal azole drugs such as fluconazole, voriconazole, and to the polyene amphotericin B.^28,29^ While no growth inhibition effect by PA was measured for the parent *C. albicans* SN152, significant growth inhibition of the *erg3ΔΔ/erg11Δ*Δ mutant was observed (Figure 1). Moreover, while *S. cerevisiae* growth in the presence of PA resumed after approximately 20 h, no *erg3ΔΔ/erg11Δ*Δ mutant cell growth was measured over the 48 h of the experiment indicating a fungicidal effect of this stilbenoid against this ergosterol-free and membrane impaired *C. albicans* strain.

We next investigated the effect of PA on *C. albicans* SC5314 and its mutant strain lacking copies of the *CHO1* gene (*cho1Δ*Δ*)* which encodes the plasma membrane-localized phosphatidylserine synthase (*cho1p*). In eukaryotic membranes, phosphatidylserine (PS) is the predominant phospholipid bearing a negative charge on the headgroup primarily due to the covalent linkage of the phosphate to serine.^30^*Cho1p* is conserved among fungi, mammals using a different pathway for the biosynthesis of this phospholipid, making it a potential pathway for antifungal targeting. Fungal cells lacking *CHO1* gene have an impaired plasma membrane that contains no PS and decreased phosphatidylethanolamine content, and were shown to be more sensitive to cell-wall-targeting CSF.^31^ While no PA-induced growth inhibition effect was observed against the parent *C. albicans* SC5314, a dose-dependent effect, like that displayed by *S. cerevisiae*, was measured for the *cho1Δ*Δ mutant. While *PA* displayed a fungicidal effect on the *erg3ΔΔ/erg11Δ*Δ mutant, it dose-dependently decreased the cell growth of the *cho1Δ*Δ strain, yet growth resumed after ∼20 h, indicative of a fungistatic effect on this impaired membrane mutant. Further investigation on the mode of action of PA in this work was constructed on *S. cerevisiae*, on whom the antifungal effect is most apparent.

### Addition of Exogenous Fungal Cell Wall Polysaccharides Attenuate PA-induced Growth Inhibition in a Dose-Dependent Manner

Previous evidence suggests that PA affects the integrity of the fungal cell wall, but it is unclear whether this is due to inhibition of one or more enzymes involved in the fungal cell wall biosynthetic machinery, direct interaction of this stilbenoid with one or more of the fungal cell wall components, or both.^20^ Generally, polysaccharides and polyphenols have been shown to interact at the molecular level, and these interactions can affect the physical properties of the polysaccharides.^32−34^ Ferulic acid, the monomer from which PA is derived, has been shown to interact with arabinan-rich pectic polysaccharides via hydrogen bonding and electrostatic forces. These interactions are diminished by elevated salt or ethanol concentrations.^35^

**Figure 2:**
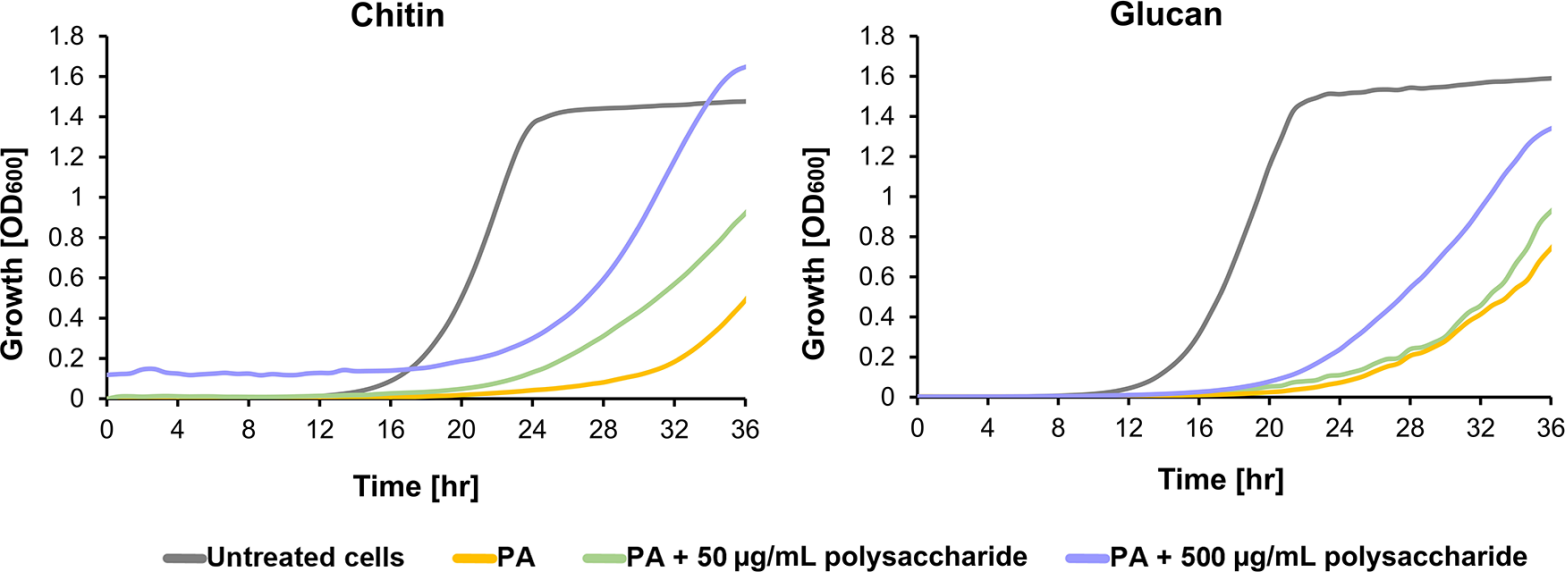
Growth of *S. cerevisiae* BY4741 cells in the presence of 512 μg/mL PA in YPD media supplemented with increasing concentrations of chitin or glucan. Growth was measured by recording the OD_600_ on an automated plate reader every 40 min for 36 h. Results were repeated in at least two independent experiments, each performed in triplicate.

To investigate whether PA inhibits *S. cerevisiae* growth by interacting with cell wall β-1,3-glucan alone or by interacting with other major cell wall polysaccharides, we investigated its effects on the growth of *S. cerevisiae* BY4741 yeast cells in the presence of elevated concentrations of pure β-glucan (96% pure glucan derived from the black yeast) or pure chitin. The results are summarized in Figure 2.

Interestingly, the growth-inhibiting effect of PA diminished in a dose-dependent manner in the presence of either glucan or chitin, with a more pronounced effect for chitin. No effect of either glucan or chitin on the growth of *S. cerevisiae* was observed at the tested concentrations (Figure S2). The interaction between PA and chitin is visually detectable, and the complex formed by this substance with chitin can be disrupted by organic solvents, as shown in Figure S4. We hypothesize that the exogenous fungal cell wall polysaccharides interact and, as a result, deflect PA from the yeast cells, reducing its free fraction in the medium and masking its interaction with the yeast cells. Several factors could cause this effect, such as binding of the exogenous polysaccharides to PA and forming complexes that are less soluble in the media or competing with the yeast cell wall polysaccharides for PA binding.

### PA Increases Lateral Yeast Cell Wall Chitin Production

In *S. cerevisiae* and other yeast, environmental stress conditions that damage the cell wall activate compensatory mechanisms to preserve cell wall integrity by remodeling its matrix.^36,37^ Arroyo and co-workers investigated the molecular basis of yeast cell responses to Congo Red and Zymolyase, two agents that induce transient cell-wall damage, by screening *S. cerevisiae* DNA microarrays. They found that genes involved in cell-wall construction and metabolism were upregulated, but the main response did not occur until hours after exposure to these agents.^38^

One of the main compensatory mechanisms for cell-wall damage caused by perturbation of β-glucan production is the enhanced production of chitin, which enhances cell-wall rigidity and the ability of the cell-wall to counter intracellular turgor pressure.^39^ Arroyo et al. found that exposure to the cell-wall-damaging agent Congo Red induced the upregulation of chitin synthase genes in *S. cerevisiae*.^38^ This suggests that chitin production is a key compensatory mechanism for cell-wall damage.

**Figure 3:**
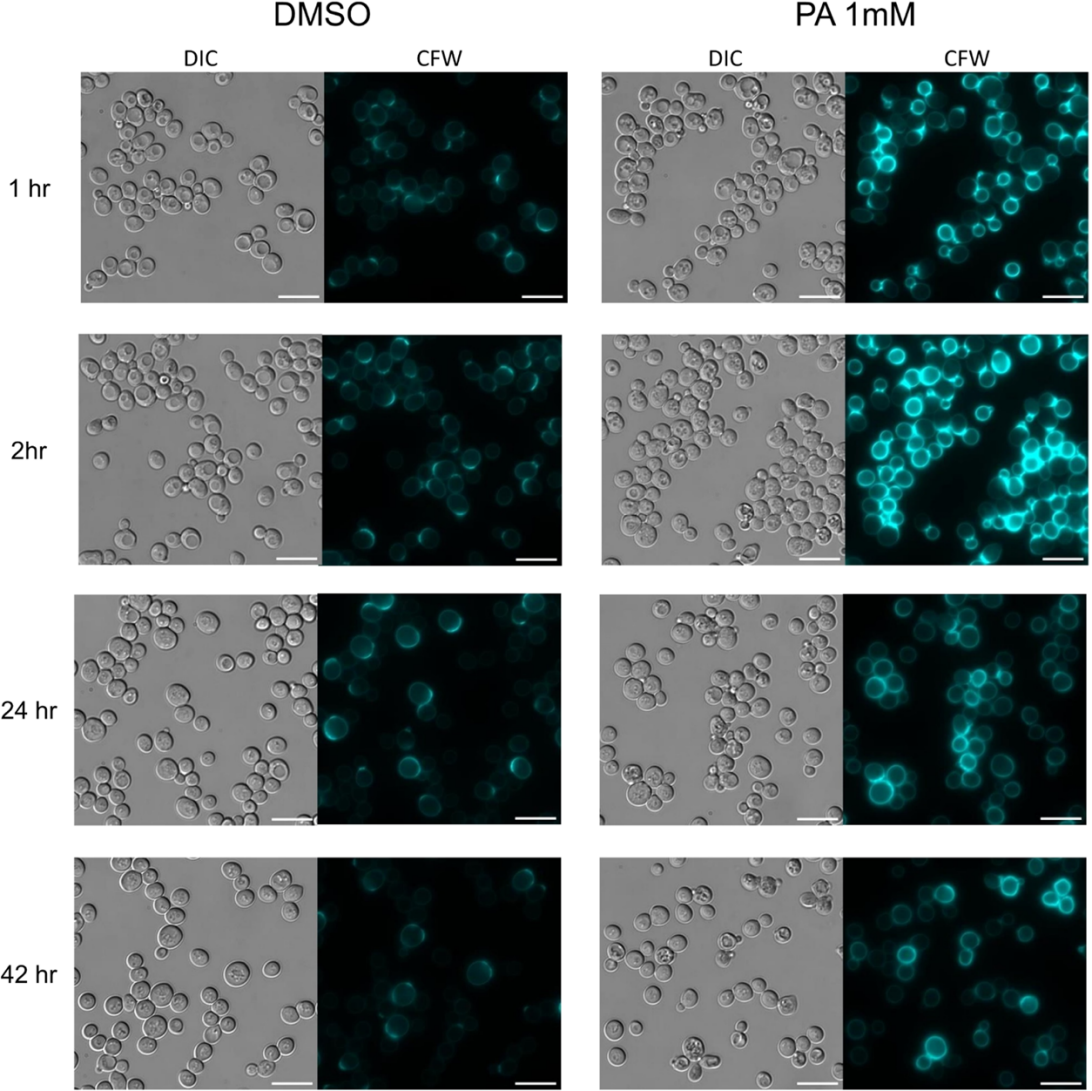
Time-dependent effect of PA on cell-wall chitin production and distribution as visualized by calcofluor white (CFW) stain. Differential interference contrast (DIC) and fluorescent images of *S. cerevisiae* (BY4741) yeast cells incubated for 1, 2, 24, or 42 h in YPD and either 1 mM PA (right) or DMSO (left). Cells were washed and stained with CFW prior to observation. Scale bars, 10 μm. A bandpass filter with an excitation of 377/50 nm and an emission wavelength of 447/60 nm was used for CFW. Similar images were obtained in at least two independent experiments.

**Figure 4:**
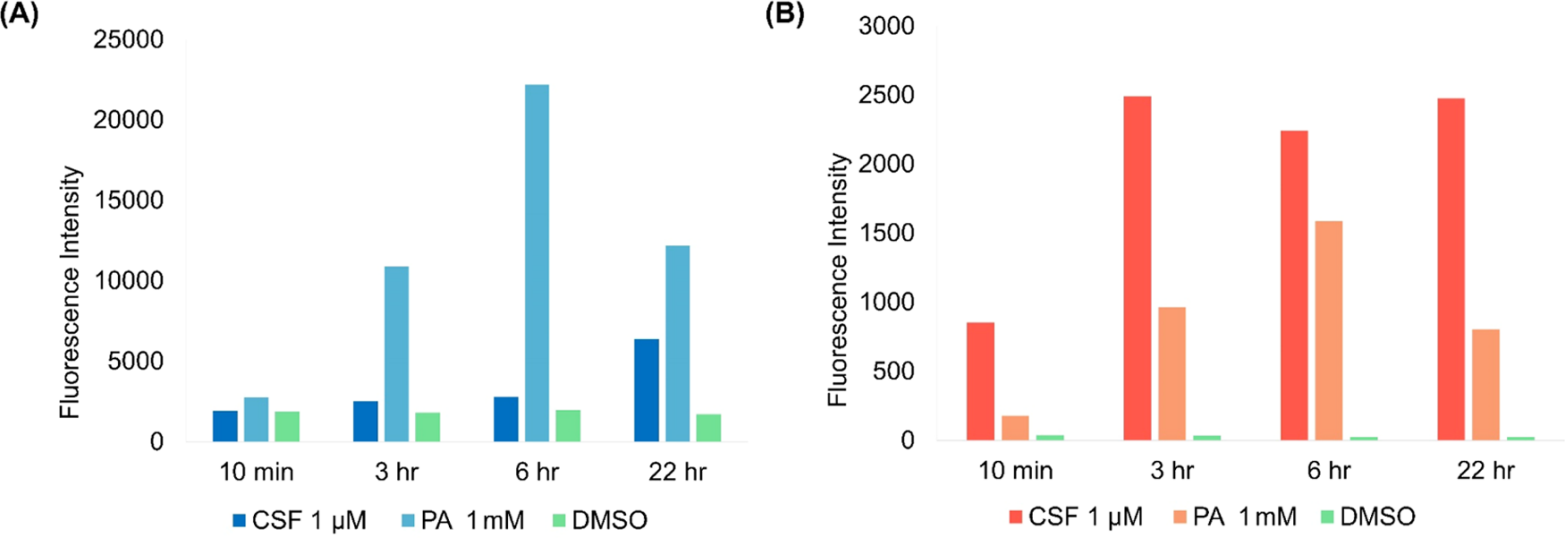
(A) Flow cytometry analysis of *S. cerevisiae* cells treated with 1 mM PA, 1 μM caspofungin (CSF), or DMSO, stained with CFW and measured over 22 h. Each value is the average of 10,000 cells measured and repeated in two independent experiments. (B) Flow cytometry of cells treated with 1 mM PA, 1 μM CSF, or DMSO, stained with PI and measured over 22 h. Each value is the average of 10,000 cells measured and repeated in two independent experiments.

To investigate if the inhibition of *S. cerevisiae* growth by PA affects cell-wall composition mechanisms, we evaluated the production of chitin in response to this stilbenoid by spatiotemporal live cell fluorescence microscopy. *S. cerevisiae* BY4741 yeast cells were incubated with 1 mM of PA (340 μg/mL) over 42 h. At several time points, a sample of cells was collected, washed in PBS, and stained with calcofluor white (CFW), a blue fluorescent dye often used to visualize cell-wall chitin in yeast cells. Interestingly, exposure to PA increased CFW signal over time compared to the vehicle-treated yeast cells, reaching a maximum at approximately 6 h after exposure (Figures 3 and 4). With longer exposure time to PA (>20 h), two yeast cell populations emerged: one population retained high CFW staining, and the second displayed reduced CFW signal and resembled that of control yeast cells (Figure 3). Furthermore, while in untreated control cells, CFW staining mainly localized to the septa region of dividing yeast cells, in PA-treated cells, CFW fluorescence increased beyond the septa regions to the entire surface of the cells in a uniform manner throughout the whole experiment (Figure 3).

A plausible explanation for the decrease in CFW staining after 6 h is that the yeast cell population comprises subgroups with varying responses to PA, resulting in different levels of enhanced chitin production. When inspecting CFW-stained cells after extended incubation with PA, the overall staining is reduced compared to cells observed after 6 h. This decrease may arise from lower PA concentrations due to binding with pre-existing chitin or from the dominance of a subpopulation in which the overproduction of chitin in response to PA is milder yet sufficient to overcome the effects of this stilbenoid. Finally, the observation that CFW staining extended beyond the septum regions to the entire cell surface in PA-treated cells suggests that PA might disrupt the normal localization of chitin in the cell wall. This disruption leads to an increased cell wall rigidity and resistance to damage.

Quantification of the differences in CFW staining between PA-treated and vehicle cells by flow cytometry (Figure 4A) revealed a spike in mean fluorescence intensity within 3 h of treatment, much faster and more significant than that observed with the echinocandin caspofungin, known to induce increased chitin biosynthesis as a compensatory mechanism. At its peak, the measured CFW intensity of PA-treated cells was ∼17-fold higher than that of vehicle cells, after which it decreased, in agreement with the live cell fluorescence microscopy observations. Of note, yeast cells of the *C. albicans* strain SN152, whose growth was not affected by PA, showed an increase in CFW chitin staining when treated with PA relative to vehicle-treated cells (Figure S5). Moreover, the CFW intensity did not decrease after prolonged incubation in *C. albicans* cells, as it did in *S. cerevisiae* cells.

To eliminate the possibility that the increased intensity of CFW staining was due to increased permeability caused by PA treatment, a PI assay was performed on PA-, caspofungin-, and DMSO-treated cells. As shown in Figure 4B, caspofungin rapidly increased the permeability of the yeast cells, whereas PA-treated cells took 6 h to reach maximum permeability, which was still significantly lower than that caused by caspofungin. This assay demonstrated the lack of correlation between permeability and CFW staining, supporting that the high CFW fluorescence intensities were due to the presence of higher chitin levels. This is consistent with the observations of chitin biosynthesis upregulation identified by Gow and co-workers, who showed that while caspofungin induces the chitin upregulation pathway by activating both Mkc1p and Mpk1p, PA stimulates only the latter.^20^

The results of the live cell fluorescence microscopy and flow cytometry experiments indicate that the elevation in cell-wall chitin content in response to PA treatment is likely a stress response to the perturbation of cell-wall assembly or biosynthesis. The appearance of two subpopulations of cells after prolonged exposure to PA, as evidenced by fluorescence microscopy, indicates that one subpopulation is characterized by a slow turn-on stress response mechanism. These observations are also consistent with the observed resumed growth of *S. cerevisiae* approximately 20 h after exposure to PA.

### Structure–Antifungal Activity Relationship Reveals That PA Has Low Tolerance for Chemical Modifications

**Scheme 2:**
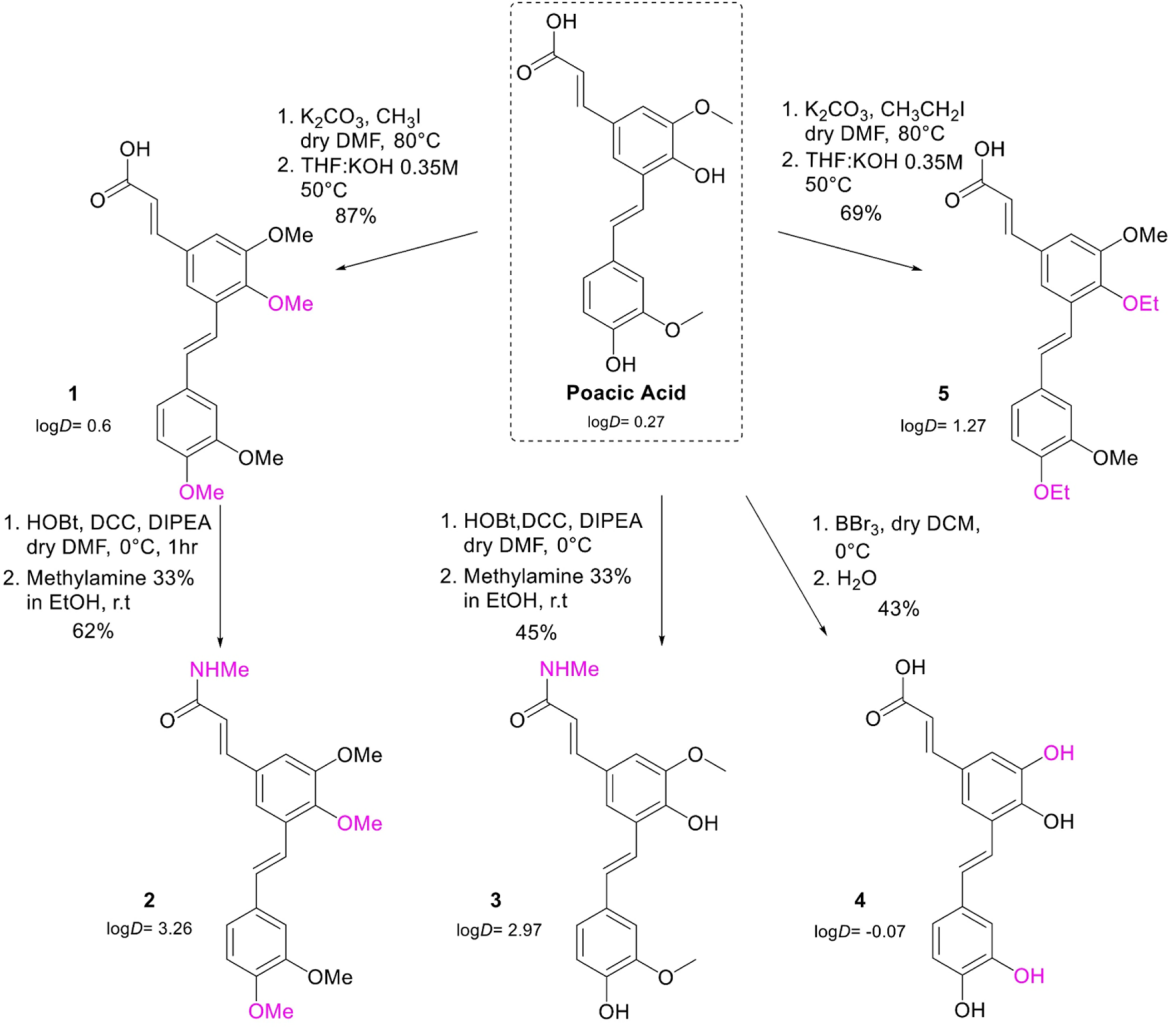
Synthesis of PA Derivatives and Their Log *D* Values

The concentration range at which PA was reported to exhibit significant antifungal activity is in the hundreds of micromolar.^14,20,21^ We therefore asked which functional groups of this stilbenoid are essential for its activity and whether this activity can be improved through chemical modifications. We prepared a collection of five PA derivatives and focused on the carboxylic acid functionality and the phenol and methoxy groups decorating this stilbenoid. We applied three types of modifications: etherification of the phenol groups, amidation of the carboxylic acid, and demethylation of the methoxy groups (Scheme 2).

Conversion of both phenol groups of PA to the corresponding methoxy or ethoxy groups (derivatives **1** and **5**, Scheme 2) increased hydrophobicity compared to the parent stilbenoid and abrogated potential phenol-based hydrogen bonds with the target (calculated Log *D* = 0.6 and 1.27, respectively). To probe the significance of PA’s carboxylic acid, methyl amide derivatives **2** and **3** were generated, neutralizing the negative charge under physiological pH and further elevating the hydrophobicity as indicated by the calculated values of the distribution coefficient (calculated Log* D* = 3.26 and 2.97, Scheme 2). Finally, demethylation of the two methoxy groups of PA afforded derivative **4** with two catechol units and increased hydrophilicity (calculated Log *D* = −0.07, Scheme 2). The structures and purity of PA and its derivatives were confirmed by analytical HPLC, ^1^H and ^13^C NMR, and HRMS (Figures S7–S34). The purity of these stilbenoids was found to be ≥95%.

**Figure 5:**
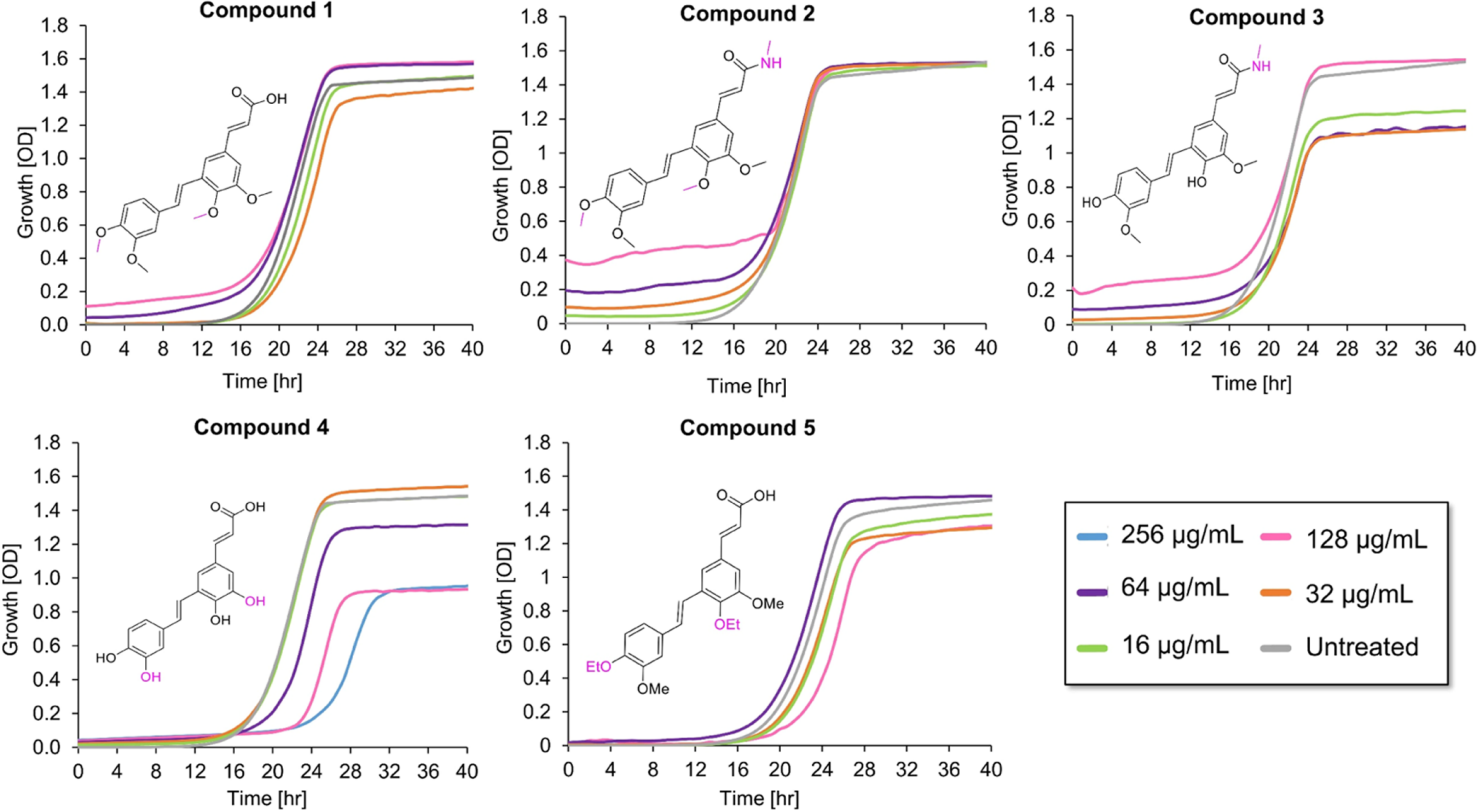
Growth of *S. cerevisiae* BY4741 cells in the presence of PA derivatives, compounds 1–5, and untreated cells in YPD media. Growth was measured by recording the OD_600_ on an automated plate reader every 40 min for 40 h. Results were repeated in at least two independent experiments, each done in triplicate.

The effect of PA and its derivatives on fungal cell growth was evaluated by the broth double-dilution assay on the *S. cerevisiae* strain BY4741. While compound **4** maintained some of the bioactivity of the parent PA, none of the remaining PA derivatives affected the growth of the tested strain, even at the maximal tested concentration, which varied depending on the solubility limitations (Figure 5). Generally, the lack of antifungal activity observed indicates that the carboxylic acid and phenol groups of PA are essential for its antifungal activity and that this stilbenoid has low tolerance for chemical modifications.

### Conjugation of PA to Echinocandins as a Cell-Wall-Directing Agent

**Figure 6:**
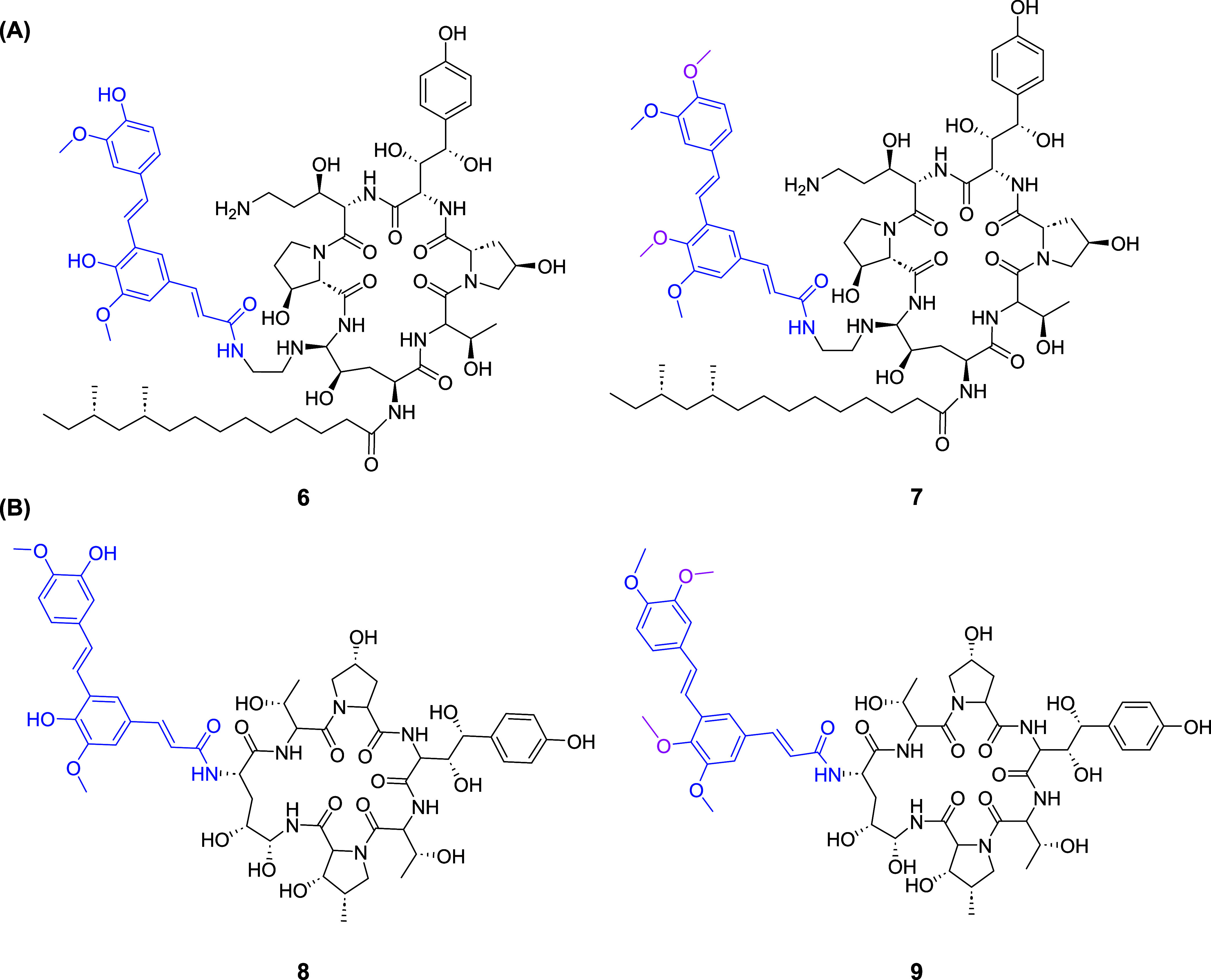
(A) Chemical structure of caspofungin conjugates with PA (**6**) or O-methylated PA (**7**). (B) Chemical structure of echinocandin B conjugates with PA (**8**) or O-methylated PA (**9**). Echinocandin core is colored black, PA blue, and PA derivation points in pink.

Through live-cell fluorescence microscopy and using fluorescent probes of echinocandin antifungal drugs, we recently provided evidence that associates the subcellular distribution of echinocandin antifungals with their efficacy. We showed that increased localization at the target-harboring cell wall resulted in higher potency.^40^ Based on this information, we hypothesized that conjugating PA, which interacts with the cell-wall components, to an echinocandin could potentially function as a cell-wall-directing moiety and place the echinocandin closer to its target, thus elevating its local concentration and potentially improving its efficacy. To test this, we selectively conjugated PA or its *O*-methylated derivative **1** to the primary amine of the ethylenediamine functionality of the echinocandin caspofungin via an amide bond (compounds **6** and **7**, Figure 6A, Scheme S1). In an additional molecular design strategy, we investigated whether PA or its derivative compound **1** could replace the hydrophobic tail of echinocandins to create a new chitin-directed echinocandin. To test this strategy, we coupled the 4,5-dihydroxyornithine of the hexapeptide echinocandin B nucleus with the carboxylic acid of either PA or compound **1** (compounds **8** and **9**, Figure 6B, Scheme S2).

We evaluated the antifungal activity of the conjugates against a panel of fungal strains using the broth double-dilution and disk diffusion assays (Table S2 and Figure S6). Conjugation of the PA unit to the intact echinocandin proved more successful; the caspofungin conjugates, compounds **6** and **7** (echinocandin–PA conjugates), displayed antifungal activity against all of the tested strains (1< MIC < 4), although their MIC values increased as compared to the parent drug. On the other hand, using PA and its methylated derivative as the hydrophobic segment of the echinocandin proved unsuccessful; the echinocandin B conjugates, compounds **8** and **9**, had no antifungal activity against any tested strains (MIC > 64).

To evaluate the interaction between the PA conjugate and cell-wall components, we measured the antifungal activity of compound **6** and caspofungin in YPD media enriched with chitin at 500 μg/mL against *S. cerevisiae* strain BY4741 (Table S2). We compared the results to those obtained in the absence of chitin. The MIC value of compound **6** increased 4-fold in the presence of exogenous chitin, while the MIC value of caspofungin remained unchanged. This increase in the MIC value suggests that the presence of this exogenous polysaccharide masks the effect of PA by directly interacting with this stilbenoid segment of compound **6**.

### Fungal Essential Metals Dose-Dependently Reduce the Antifungal Activity of PA

Ferulic acid, from which PA is derived, is a natural metal chelator that has been shown to protect mice brains from the side effects of iron overload.^41,42^ We therefore hypothesized that PA may interfere with metal homeostasis in the range of its measurable antifungal activity against *S. cerevisiae* strains. This activity could affect yeast growth and contribute to the antifungal activity of this stilbenoid. Four main metals are essential for fungi: copper, iron, zinc, and manganese.^43^ In fungal cells, iron serves as a cofactor in the form of heme and iron–sulfur clusters, which are key to numerous cellular processes.^44,45^ Copper is essential for the activation of metalloproteins such as superoxide dismutase and cytochrome c oxidase, and it serves as an essential component of iron–sulfur clusters.^46,47^ Close to 5% of the fungal proteome is composed of zinc-binding proteins, and approximately 8% of yeast genomes correlate to zinc-binding proteins. In *S. cerevisiae*, a high percentage of the zinc-binding proteins are related to DNA binding, regulation of transcription, transcription factor activity, and stimuli responses.^48,49^ Finally, fungi have evolved complex regulatory systems to acquire, distribute, and utilize manganese. Disruption of manganese homeostasis in pathogenic fungi leads to severe phenotypes and reduces or abrogates virulence.^43^

**Figure 7:**
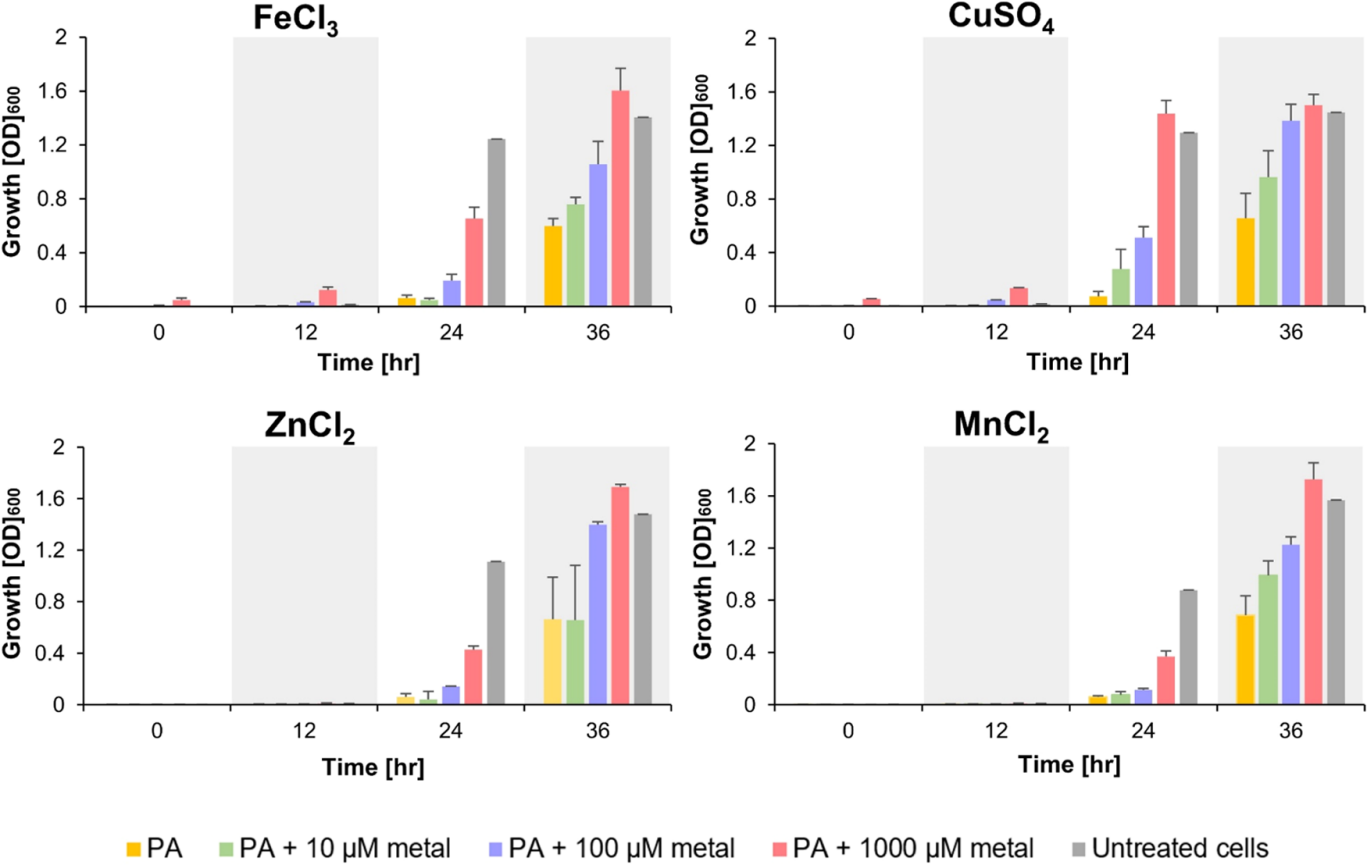
*S. cerevisiae* BY4741 growth in the presence of 512 μg/mL PA in media supplemented with metal ions at increasing concentrations. Untreated cells grown in YPD media were used as a control. Growth was measured by recording the OD_600_ values on an automated plate reader every 40 min for 36 h. Results are presented at 12 h points and were averaged from at least two independent experiments each done in triplicate.

To investigate whether and to what extent metal chelation by PA contributes to its growth-inhibiting effect, we determined its activity in the presence of increasing concentrations of exogenous iron, copper, zinc, or manganese ions in *S. cerevisiae* cells. The results are summarized in Figure 7.

A dose-dependent reduction in the effect of PA on growth was observed for all four metals, with the effect being more pronounced for copper and zinc and the least pronounced for manganese (Figure 7). At 10 μM, the lowest tested concentration, copper increased growth in the presence of PA by approximately 260% relative to cells treated with PA alone after 24 h of treatment. Under normal growth conditions for yeast, the concentration of copper ranges between 1 and 10 μM. Control cultures of *S. cerevisiae* cells were unaffected when grown in the presence of these metal ions (Figure S3). Since PA exerts its cell-growth-inhibiting effect at a high concentration range, these results support that this stilbenoid affects essential metal homeostasis, which can contribute, at least in part, to its inhibitory effect.

## Conclusions

This study has unveiled new insights into poacic acid (PA), a plant-derived antifungal stilbenoid with potential applications. PA exhibits fungistatic properties against *S. cerevisiae* yeast but no significant effect against *Candida*, except for strains with compromised plasma membrane structures. Structure–activity relationship studies emphasize the importance of PA’s carboxylic acid and phenol groups for antifungal activity, highlighting its low tolerance for chemical modifications. To investigate PA’s potential as a cell-wall directing agent to enhance echinocandin antifungals’ efficacy, we synthesized and evaluated two types of conjugates: PA conjugated to caspofungin’s ethylenediamine functionality, and PA conjugated to the echinocandin B nucleus. While echinocandin B conjugates lacked antifungal activity, caspofungin conjugates displayed activity across all strains, albeit with increased MIC values compared to those of the parent drug. The optimal conjugation strategy warrants further investigation.

Exposure to PA increased the level of chitin production in a time-dependent manner, altering its localization within yeast cells. PA-induced growth inhibition was mitigated in the presence of exogenous β-glucan or chitin, particularly the latter, suggesting that PA interferes with cell-wall integrity and function, resulting in chitin accumulation. Our results indicate that the interaction between PA and the increasing amount of chitin, which occurs as a stress response to this stilbenoid, dilutes its antifungal effect over time.

PA’s growth-inhibiting effect on *S. cerevisiae* is also attenuated by exogenous metal ions, with copper and iron exhibiting greater efficacy than zinc and manganese. This underscores PA’s potential metal-chelating mechanism affecting metal homeostasis.

This study’s findings indicate that PA, like many flavonoid and stilbenoid phytochemicals, exerts its antifungal activity by affecting multiple cellular processes and likely more than one target. While its suitability for clinical antifungal development is uncertain, PA holds promise as an eco-friendly antifungal agent for use in industries and agriculture, where there is a pressing demand for easily producible and novel antifungals.

## Materials and Methods

### Chemistry

^1^H NMR spectra were recorded on a Bruker Avance 400 or 500 MHz spectrometer. ^13^C NMR spectra were recorded on a Bruker Avance 400 or 500 MHz spectrometer at 100 and 125 MHz. Chemical shifts (reported in ppm) were calibrated to CD_3_OD (^1^H: δ = 3.31, ^13^C: δ = 49.0) or DMSO-*d*_6_ (^1^H: δ = 2.50, ^13^C: δ = 39.52). Multiplicities are reported using the following abbreviations: s = singlet, d = doublet, t = triplet, dd = doublet of doublets, ddd = doublet of doublet of doublets, td = triplet of doublets, and m = multiplet. Coupling constants (J) are given in Hertz. High-resolution electrospray ionization (HRESI) mass spectra were measured on a Waters Xevo G2 XS QTOF instrument. HR-APPI MS was measured on a Waters Synapt QTOF. Low-resolution electrospray ionization mass spectra (ESI-MS) were measured on a Waters Acquity SQD-2 system. Chemical reactions were monitored by TLC (Merck, Silica gel 60 F254), and visualization was achieved using a UV lamp. All chemicals, unless otherwise stated, were obtained from commercial sources, including chitin (CAS 1398–61–4) and glucan (96% pure glucan derived from black yeast, CAS 9012–72–0). CFW (1 mg/mL) was purchased from Sigma-Aldrich. Compounds were purified using Geduran Si 60 chromatography (Merck). The preparative reversed-phase high-pressure liquid chromatography (RP-HPLC) system used was an ECOM system equipped with a 5-μm, C-18 Phenomenex Luna Axia column (250 mm × 21.2 mm). Analytical RP-HPLC was performed on a VWR Hitachi instrument equipped with a diode array detector and an Alltech Apollo C18 reversed-phase column (5 μm, 4.6 mm × 250 mm). The flow rate was 1 mL/min. Solvent A was 0.1% TFA in water; solvent B was acetonitrile. The SpectraMax i3x Platform spectrophotometer from Molecular Devices was used for fluorescence measurements. Log *D* values were calculated using MarvinSketch (ver. 6.3.1) with default parameters and with an electrolyte concentration of 0.1 M NaCl at physiological pH (7.4).

### Synthesis of Poacic Acid (PA)

PA was synthesized as previously reported by Ralph et al. with minor modifications as follows: To a stirred solution of ferulic acid (5 g, 25.8 mmol) dissolved in absolute ethanol (50 mL) was slowly added acetyl chloride (3 mL) was slowly added. After 48 h, the volatiles were removed under vacuum at 40 °C. Ethyl ferulate was purified via column chromatography (elution with 20% ethyl acetate in petroleum ether; yield 91%). Ethyl ferulate (4.5 g) was dissolved in 90 mL of acetone and diluted with 270 mL of deionized water. Urea–H_2_O_2_ complex (1.05 g) dissolved in 7.5 mL of double-distilled water (ddH_2_O) was added, followed immediately by the addition of horseradish peroxidase (2.05 mg) dissolved in 5 mL of ddH_2_O. The reaction mixture was diluted to 510 mL and stirred with a magnetic stirrer for approximately 45 min. Upon completion (disappearance of ethyl ferulate by TLC analysis), the reaction mixture was acidified with HCl (6 M, 3 mL) to pH < 3. Acetone was removed under vacuum, and the remaining liquid was filtered off. The crude solids were dissolved in 160 mL of 0.35 M NaOH and heated to 90 °C for 18 h. Thin-layer chromatography (TLC) and mass spectrometry (MS) analysis were used to follow the consumption of crude differulates and formation of hydrolyzed ([M-H]- *m*/*z* 385.2) and decarboxylated ([M-H] *m*/*z* 341.1) products. PA was purified by column chromatography (elution with 30% mixture of ethyl acetate/ethanol/acetic acid (90:10:1 v/v/v) in petroleum ether). Reversed-phase high-pressure liquid chromatography (RP-HPLC) (mobile phase: acetonitrile in water (containing 0.1% TFA), gradient from 10 to 90%; flow rate: 20 mL/min) afforded pure PA (6% overall yield). NMR Spectra were in accordance with the literature.

#### Compound **1**

PA (83 mg, 0.24 mmol, 1 equiv) was dissolved in dry DMF (3 mL) under argon. Potassium carbonate (160 mg, 1.16 mmol, 5 equiv) was added, followed by methyl iodide (300 μL, 4.15 mmol, 20 equiv). The reaction was heated to 80 °C for 5 h, as indicated by TLC analysis showing complete consumption of PA. DMF was evaporated to a minimum under vacuum, and the crude oil was diluted in 10 mL of EtOAc and washed with 10 mL portions of brine. The aqueous phase was extracted twice with EtOAc (10 mL) and brine, and the organic phase was then purified by column chromatography (elution with 25% EtOAc in PE). Methylated PA was then dissolved in 3 mL of THF, and potassium hydroxide (0.5 M, 4 mL) was added. The reaction was heated to 50 °C for 3 h. Upon completion, the reaction was acidified with HCl (1 M, 3 mL) to pH < 1. Compound 1 was extracted with EtOAc, washed with brine, and dried over MgSO_4_, and the solvent was reduced under vacuum to yield 77 mg (87% yield) of pure compound 1 as a light-yellow powder. HRESI-MS *m*/*z* calculated for C_21_H_22_O_6_, 369.1338; found [M – H]^−^, 369.1348. ^1^H NMR (400 MHz, DMSO-*d*_6_) δ 12.34 (s, 1H), 7.64 (d, *J* = 1.7 Hz, 1H), 7.57 (d, *J* = 15.9 Hz, 1H), 7.33 (d, *J* = 16.5 Hz, 1H), 7.29 (d, *J* = 1.7 Hz, 1H), 7.19–7.23 (m, 2H), 7.18–7.19 (m, 1H), 7.14 (dd, *J* = 8.3, 1.9 Hz, 1H), 6.97 (d, *J* = 15.9 Hz, 1H), 3.87 (s, 3H), 3.83 (s, 3H), 3.79 (s, 3H), 3.78 (s, 3H). ^13^C NMR (100 MHz, DMSO-*d*_6_) δ: 167.8, 152.9, 148.9, 147.6, 143.9, 131.1, 130.6, 130.3, 130.0, 119.8, 119.5, 118.7, 118.4, 111.9, 110.5, 109.6, 60.6, 55.9, 55.5.

#### Compound **2**

Compound 1 (20 mg, 0.054 mmol, 1 equiv) was dissolved in dry DMF (0.8 mL). HOBt (22 mg, 0.162 mmol, 3 equiv) and DCC (33 mg, 0.26 mmol, 3 equiv) were added to the reaction flask under argon and cooled to 0 °C. DIPEA (46 μL, 0.27 mmol, 5 equiv) was added, and the reaction was stirred at 0 °C for 1 h. Methylamine 33% in EtOH (20 μL, 0.162 mmol, 3 equiv) was dissolved in 0.3 mL of dry DMF and added dropwise to the reaction mixture at 0 °C. The reaction was stirred for 10 min, then brought to room temperature, and stirred overnight. Reaction progress was followed by TLC and MS analysis. RP-HPLC (mobile phase: acetonitrile in water (containing 0.1% TFA), gradient from 10% to 90%; flow rate: 20 mL/min) afforded pure compound 2 (12.8 mg, 62% yield) as a yellow powder. HRESI-MS *m*/*z* calculated for C_20_H_25_NO_5_Na, 406.1630; found [M + Na]^+^, 406.1623. ^1^H NMR (400 MHz, DMSO-*d*_6_) δ 12.34 (s, 1H), 7.64 (d, *J* = 1.7 Hz, 1H), 7.57 (d, *J* = 15.9 Hz, 1H), 7.33 (d, *J* = 16.5 Hz, 1H), 7.29 (d, *J* = 1.7 Hz, 1H), 7.19–7.23 (m, 1H), 7.18–7.19 (m, 1H), 7.14 (dd, *J* = 8.3, 1.9 Hz, 1H), 6.97 (d, *J* = 15.9 Hz, 1H), 3.87 (s, 3H), 3.83 (s, 3H), 3.79 (s, 3H), 3.78 (s, 3H). ^13^C NMR (100 MHz, DMSO-*d*_6_) δ 167.8, 152.9, 148.9, 147.6, 143.9, 131.1, 130.6, 130.3, 130.0, 119.8, 119.5, 118.7, 118.4, 111.9, 110.5, 109.6, 60.6, 55.9, 55.5.

#### Compound **3**

PA (22 mg, 0.064 mmol, 1 equiv) was dissolved in dry DMF (0.7 mL) under argon. HOBt (27.6 mg, 0.20 mmol, 3 equiv) and DCC (40 mg, 0.19 mmol, 3 equiv) were added to the reaction flask and cooled to 0 °C. DIPEA (57 μL, 0.33 mmol, 5 equiv) was added, and the reaction was stirred at 0 °C for 1 h. Methylamine 33% in EtOH (24 μL, 0.19 mmol, 3 equiv) was dissolved in 0.7 mL of dry DMF and added dropwise to the reaction mixture at 0 °C. The reaction was stirred for 10 min, then brought to room temperature and stirred overnight. The reaction was followed by TLC and MS analysis. RP-HPLC (mobile phase: acetonitrile in water (containing 0.1% TFA), gradient from 10% to 90%; flow rate: 20 mL min^–1^) afforded pure compound 4 (10.2 mg, 45% yield) as a white powder. HRESI-MS *m*/*z* calculated for C_20_H_22_NO_5_, 356.1498; found [M + H]^+^, 356.1492. ^1^H NMR (400 MHz, DMSO-*d*_6_) δ 9.27 (s, 1H), 9.13 (s, 1H), 7.90 (q, *J* = 4.6 Hz, 1H), 7.39 (d, *J* = 1.7 Hz, 1H), 7.36 (d, *J* = 15.7 Hz, 1H), 7.21 (d, *J* = 16.3 Hz, 1H), 7.14 (d, *J* = 16.3 Hz, 1H), 7.12 (s, 1H), 7.05 (d, *J* = 1.8 Hz, 1H), 6.96 (dd, *J* = 8.2, 1.8 Hz, 1H), 6.77 (d, *J* = 8.2 Hz, 1H), 6.50 (d, *J* = 15.7 Hz, 1H), 3.86 (s, 3H), 3.83 (s, 3H), 2.70 (d, *J* = 4.6 Hz, 3H). ^13^C NMR (100 MHz, DMSO-*d*_6_) δ: 165.9, 148.0, 147.8, 146.6, 145.1, 138.9, 129.2, 129.0, 126.0, 124.6, 119.9, 119.7, 119.4, 118.5, 115.6, 106.7, 108.4, 55.9, 55.5, 25.7.

#### Compound **4**

PA (30 mg, 0.088 mmol, 1 equiv) was dissolved in dry DCM (3 mL) under argon and cooled to 0 °C. BBr_3_ (50 μL, 0.52 mmol, 6 equiv) was added dropwise, and the solution became clear brown. Dry DCM (3 mL) was added, and the reaction was stirred at room temperature for 2 h. The reaction progress was monitored by TLC and MS analysis. Upon completion, the reaction solution was poured dropwise onto ice. DCM was removed by vacuum, and the product was extracted from the aqueous phase with two 20 mL portions of EtOAc and then dried over MgSO_4_. RP-HPLC was performed (mobile phase: acetonitrile in H_2_O (containing 0.1% TFA), gradient from 30% to 70%; flow rate: 20 mL min^–1^) to achieve pure compound 4 (12 mg, 43% yield). MS: (APPI in NEG mode with toluene as photosensitizer) *m*/*z* calculated for C_17_H_13_O_6_, 313.0712; found [M – H]^−^, 313.0716. ^1^H NMR (400 MHz, CD_3_OD) δ 7.58 (d, *J* = 15.9 Hz, 1H), 7.27 (d, *J* = 1.9 Hz, 1H), 7.23 (d, *J* = 16.5 Hz, 1H), 7.09 (d, *J* = 16.5 Hz, 1H), 7.05 (d, *J* = 2.0 Hz, 1H), 6.96 (d, *J* = 1.9 Hz, 1H), 6.90 (dd, *J* = 8.2, 1.9 Hz, 1H), 6.76 (d, *J* = 8.2 Hz, 1H), 6.28 (d, *J* = 15.9, 1H). ^13^C NMR (100 MHz, CD_3_OD) δ: 171.2, 147.1, 147.0, 146.8, 146.5, 131.7, 130.5, 127.2, 126.6, 121.1, 120.2, 116.4, 116.0, 113.8, 112.3.

#### Compound **5**

PA (85 mg, 0.25 mmol, 1 equiv) was dissolved in dry DMF (3 mL) under argon. Potassium carbonate (140 mg, 0.99 mmol, 5 equiv) was added, followed by ethyl iodide (160 mg, 1.16 mmol, 4 equiv), and the reaction was heated to 80 °C for 5 h. TLC analysis showed complete consumption of PA. DMF was evaporated by vacuum, and the crude oil was extracted with 10 mL of EtOAc and washed with 10 mL portions of brine. The aqueous phase was extracted twice with EtOAc (10 mL) and brine, evaporated under vacuum, and then purified by column chromatography (elution at 25% EtOAc in PE). Ethylated PA was then dissolved in 3 mL of THF, potassium hydroxide (0.5 M, 4 mL) was added, and the reaction was heated to 50 °C for 6 h. Upon completion, the reaction was acidified with HCl (1M, 3 mL) to pH < 1. Compound 5 was extracted with EtOAc, washed with brine, and dried over MgSO_4_, and the solvent was reduced under vacuum to achieve 60.1 mg (69% yield). HRESI-MS *m*/*z* calculated for C_23_H_26_O_6_Na, 421.1627; found [M + Na]^+^, 421.1621. ^1^H NMR (400 MHz, DMSO-*d*_6_) δ 12.34 (s, 1H), 7.64 (d, *J* = 1.6 Hz, 1H), 7.57 (d, *J* = 15.9 Hz, 1H), 7.32 (d, *J* = 16.6 Hz, 1H), 7.28 (d, *J* = 1.7 Hz, 1H), 7.23 (d, *J* = 16.6 Hz, 1H), 7.15 (d, *J* = 1.9 Hz, 1H), 7.10 (dd, *J* = 8.3, 1.9 Hz, 1H), 6.96 (d, *J* = 8.4 Hz, 1H), 6.4 (d, *J* = 15.9 Hz, 1H), 3.99–4.05 (m, 4H), 3.86 (s, 3H), 3.82 (s, 3H), 1.31–1.35 (m, 6H). ^13^C NMR (100 MHz, DMSO-*d*_6_) δ: 167.8, 153.1, 149.0, 148.1, 146.6, 143.9, 131.4, 130.3, 130.1, 130.0, 119.8, 119.6, 118.6, 118.3, 112.9, 110.4, 109.6, 68.7, 63.7, 55.9, 55.4, 15.5, 14.7.

#### PA-NHS

PA (40 mg, 0.116 mmol, 1 equiv) was dissolved in 1 mL of dry DMF under argon. DMAP (70 mg, 0.58 mmol, 5 equiv) and TEA (82 μL, 0.58 mmol, 5 equiv) were added. Disuccinimidyl carbonate (30 mg, 0.116 mmol, 1 equiv) was added, and the reaction progress was followed by TLC analysis (50% EtOAc/EtOH/AcOH 90:10:1 in PE). The reaction was stirred overnight. Upon completion, the reaction was quenched with acetic acid (53 μL, 1 mmol), and solvents were evaporated to minimum via vacuum. The crude mixture was separated via RP-HPLC (mobile phase: Acetonitrile in H_2_O (containing 0.1% TFA), gradient from 10% to 90%; flow rate: 20 mL/min) to afford N-hydroxysuccinimide ester of PA (16 mg, 31% yield). ESI-MS *m*/*z* was calculated for C_23_H_20_NO_8_ and found [M – H]^−^, 438.5.

#### Compound 1-NHS

Compound 1-NHS was prepared by the same procedure as PA-NHS with the following amounts: compound 1 (38.7 mg, 0.1 mmol, 1 equiv), DMAP (64 mg, 0.52 mmol, 5 equiv), DSC (26.7 mg, 0.1 mmol, 1 equiv), acetic acid (26 μL, 0.5 mmol). ESI-MS *m*/*z* was calculated for C_23_H_20_NO_8_Na found [M + Na]^+^, 460.6.

#### Compound **6**

Caspofungin diacetate (48.5 mg, 0.04 mmol, 2 equiv) and TEA (5.5 μL, 2 equiv) were dissolved in dry DMF (2 mL) under argon and treated with PA-NHS (9 mg, 0.02 mmol, 1eq.). The reaction was stirred at ambient temperature for 3 h. Reaction progress was monitored by ESI-MS following the disappearance of PA-NHS. Upon completion, the solvent was removed under vacuum, and the residue was purified by preparative RP-HPLC (mobile phase: Acetonitrile in H_2_O (containing 0.1% TFA), gradient from 10% to 90%; flow rate: 20 mL/min) to afford pure compound 6 (14 mg, 50% yield). HRESI-MS *m*/*z* calculated for C_71_H_105_N_10_O_20_, 1417.7507; found [M + H]^+^, 1417.7507. ^1^H NMR (500 MHz, CD_3_OD) δ 7.56 (d, *J* = 15.6 Hz, 1H), 7.37 (d, *J* = 1.6 Hz, 1H), 7.28 (d, *J* = 16.5 Hz, 1H), 7.10–7.14 (m, 4H), 7.04 (d, *J* = 1.6 Hz, 1H), 6.97 (dd, *J* = 8.2, 1.8 Hz, 1H), 6.79 (d, *J* = 4.1 Hz, 1H), 6.76 (d, *J* = 8.6 Hz, 2H), 6.51 (d, *J* = 15.6 Hz, 1H), 5.36 (s, 1H), 5.00 (d, *J* = 3.2 Hz, 1H), 4.90–4.92 (m, 1H), 4.52–4.64 (m, 4H), 4.29–4.35 (m, 4H), 4.21 (dd, *J* = 1.6, 8.1 Hz, 1H), 4.02–4.06 (m, 1H), 3.97–3.99 (m, 1H), 3.93 (s, 3H), 3.91 (s, 3H), 3.84–3.89 (m, 2H), 3.77–3.82 (m, 2H), 3.45–3.50 (m, 1H), 3.12–3.22 (m, 2H), 3.01–3.12 (m, 2H), 2.43–2.47 (m, 1H), 2.24–2.33 (m, 3H), 2.17–2.21 (m, 2H), 1.99–2.11 (m, 3H), 1.80–1.87 (m, 1H), 1.52–1.61 (m, 2H), 1.14–1.32 (m, 24H), 1.12–1.09 (m, 1H), 0.78–0.88 (m, 10H). ^13^C NMR (125 MHz, CD_3_OD) δ 176.7, 174.7, 173.5, 173.3, 172.9, 172.8, 172.7, 171.1, 169.0, 158.6, 149.5, 149.2, 147.8, 147.6, 143.9, 132.9, 131.4, 130.7, 129.5, 127.0, 126.2, 121.4, 121.1, 120.9, 117.5, 116.3, 116.2, 110.2, 108.9, 77.4, 75.5, 75.0, 72.6, 71.3, 68.4, 68.3, 67.4, 64.6, 62.8, 58.2, 57.1, 56.6, 56.4, 56.3, 56.2, 56.0, 50.3, 47.1, 46.8, 45.8, 39.1, 38.4, 38.0, 37.7, 37.0, 35.4, 34.7, 32.8, 31.2, 30.8, 30.7, 30.6, 30.4, 30.2, 28.0, 27.0, 20.6, 20.2, 19.9, 11.5.

#### Compound **7**

Caspofungin diacetate (41.5 mg, 0.034 mmol, 2 equiv) and TEA (4.7 μL, 2 equiv) were dissolved in dry DMF (2 mL) under argon and treated with NHS ester of compound 1 (8 mg, 0.02 mmol, 1eq.). The reaction was stirred at ambient temperature for 3 h. Reaction progress was monitored by ESI-MS following the disappearance of the NHS ester. Upon completion, the solvent was removed under vacuum and the residue was purified by preparative RP-HPLC (mobile phase: Acetonitrile in H_2_O (containing 0.1% TFA), gradient from 10% to 90%; flow rate: 20 mL/min) to afford pure compound 7 (11.6 mg, 47% yield). HRESI-MS *m*/*z* calculated for C_73_H_109_N_10_O_20_, 1445.7820; found [M + H]^+^, 1445.7828. ^1^H NMR (400 MHz, CD_3_OD) δ 9.11 (d, *J* = 8.4, 1H), 7.61 (d, *J* = 15.6 Hz, 1H), 7.52 (d, *J* = 9.0 Hz, 1H), 7.47 (d, *J* = 1.6 Hz, 1H), 7.31 (d, *J* = 16.5 Hz, 1H), 7.13–7.20 (m, 5H), 6.98 (d, *J* = 8.4 Hz, 1H), 6.78 (d, *J* = 8.6 Hz, 2H), 6.63 (d, *J* = 15.6 Hz, 1H), 5.41 (s, 1H), 5.02 (d, *J* = 3.3 Hz, 1H), 4.92–4.95 (m, 1H), 4.54–4.66 (m, 4H), 4.31–4.37 (m, 4H), 4.22 (dd, *J* = 8.0, 1.5 Hz, 1H), 4.09–4.05 (m, 1H), 3.98–4.01 (m, 1H), 3.94 (s, 3H), 3.92 (s, 3H), 3.89 (s, 3H), 3.88 (s, 3H), 3.82–3.85 (m, 2H), 3.49–3.53 (m, 1H), 3.15–3.23 (m, 2H), 3.05–3.12 (m, 2H), 2.45–2.49 (m, 1H), 2.30–2.34 (m, 3H), 2.19–2.26 (m, 2H), 2.01–2.13 (m, 3H), 1.82–1.91 (m, 1H), 1.55–1.59 (m, 2H), 1.10–1.41 (m, 24H), 1.01–1.10 (m, 1H), 0.79–0.92 (m, 10H). ^13^C NMR (100 MHz, CD_3_OD) δ 176.8, 176.7, 174.7, 173.6, 173.5, 173.4, 172.9, 172.7, 170.6, 169.0, 158.6, 154.8, 150.8, 150.7,149.7, 143.0, 133.2, 132.9, 132.1, 131.9, 131.7, 129.5, 121.4, 121.3, 120.0, 119.8, 116.2, 113.0, 110.9, 110.7, 77.5, 75.5, 75.0, 72.6, 71.3, 68.4, 68.3, 67.4, 64.7, 62.8, 61.4, 58.3, 57.1, 56.6, 56.5, 56.4, 56.3, 56.0, 50.3, 47.1, 46.6, 48.9, 39.1, 38.4, 38.0, 37.6, 37.0, 35.4, 34.7, 32.8, 31.2, 30.9, 30.7, 30.6, 30.5, 30.3, 28.0, 27.0, 20.6, 20.6, 20.2, 19.9, 11.5.

#### Compound **8**

PA (16.4 mg, 0.048 mmol, 2 equiv) was dissolved in dry DMF (0.6 mL) under argon and cooled to 0 °C. HOBt (10.3 mg, 0.0768 mmol, 3.2 equiv) and DCC (10 mg, 0.048 mmol, 2 equiv) were added and stirred for 1 h. Echinocandin B (20 mg, 0.024 mmol, 1 equiv) was dissolved in DMF (0.5 mL) and added dropwise to the reaction where it was stirred for 10 min at 0 °C and then at room temperature for 24 h. The reaction progress was followed by MS. Upon full consumption of the reactant, crude was separated by RP-HPLC (mobile phase: Acetonitrile in H_2_O (containing 0.1% TFA), gradient from 30% to 70%; flow rate: 20 mL/min) to afford pure compound 8 (10.1 mg, 37% yield). HRESI-MS *m*/*z* calculated for C_53_H_67_N_7_O_20_Na, 1144.4339; found [M + Na]^+^, 1144.4352. ^1^H NMR (400 MHz, DMSO-*d*_6_) δ 9.32 (s, 1H), 8.12–8.15 (m, 2H), 8.02 (d, *J* = 9.0 Hz, 1H), 7.40 (d, *J* = 1.3 Hz, 1H), 7.35 (d, *J* = 15.6 Hz, 1H), 7.29 (d, *J* = 8.8 Hz, 1H), 7.21 (d, *J* = 16.5 Hz, 1H), 7.09–7.15 (m, 2H), 7.06 (d, *J* = 1.3, 1H), 7.02 (d, *J* = 8.6, 2H), 6.97 (dd, *J* = 8.2, 1.7 Hz, 1H), 6.77 (d, *J* = 8.2, 1H), 6.60–6.70 (m, 3H), 5.00 (dd, *J* = 9.3, 2.8 Hz, 1H), 4.75 (m, 1H), 4.67 (dd, *J* = 9.2, 4.6 Hz, 1H), 4.41 (m, 1H), 4.36–4.37 (m, 3H), 4.22 (d, *J* = 1.44 Hz, 1H), 4.15 (d, *J* = 7.9 Hz, 1H), 3.9–4.05 (m, 5H), 3.87 (s, 3H), 3.82 (s, 3H), 3.67–3.9 (m, 4H), 3.19 (t, *J* = 9.6 Hz, 1H), 2.32–2.40 (m, 1H), 2.17–2.26 (m, 1H), 1.82–1.95 (m, 2H), 1.65–1.75 (m, 1H), 1.10 (d, *J* = 6.2 Hz, 6H), 0.96 (d, *J* = 6.8 Hz, 3H). ^13^C NMR (100 MHz, DMSO-*d*_6_) δ 172.1, 171.2, 170.9, 170.3, 169.8, 168.6, 165.5, 158.2, 156.9, 148.4, 148.2, 146.9, 145.5, 139.9, 132.9, 129.7, 129.5, 128.6, 126.5, 125.0, 120.3, 120.2, 119.0, 116.0, 115.1, 110.2, 108.9, 75.8, 73.6, 72.9, 72.8, 69.6, 68.8, 68.2, 66.6, 61.3, 57.2, 56.3, 56.2, 56.0, 55.6, 54.7, 51.7, 50.1, 37.7, 35.0,19.7, 11.3.

#### Compound **9**

Compound 1 (17.8 mg, 0.048 mmol, 2 equiv) was dissolved in dry DMF (0.6 mL) under argon and cooled to 0 °C. HOBt (10.3 mg, 0.076 mmol, 3.2 equiv) and DCC (10 mg, 0.048 mmol. 2 equiv) were added and stirred for 1 h. Echinocandin B (20 mg, 0.024 mmol, 1 equiv) was dissolved in DMF (0.5 mL) and added dropwise to the reaction where it was stirred for 10 min at 0 °C and then at room temperature for 24 h. Reaction progress was followed by MS. Upon full consumption of reactant, crude was separated by RP-HPLC (mobile phase: Acetonitrile in H_2_O (containing 0.1% TFA), gradient from 30% to 70%; flow rate: 20 mL/min) to afford pure compound 9 (14.8 mg, 54% yield). HRESI-MS *m*/*z* calcd for C_55_H_71_N_7_O_20_Na, 1172.4652; found [M + Na]^+^, 1172.4670. ^1^H NMR (400 MHz, DMSO-*d*_6_) δ 9.31 (s, 1H), 8.23 (d, *J* = 7.9 Hz, 1H), 8.14 (d, *J* = 7.2 Hz, 1H), 8.00 (d, *J* = 9.0 Hz, 1H), 7.50 (s, 1H), 7.39 (d, *J* = 15.7 Hz, 1H), 7.31 (d, *J* = 9.2 Hz, 1H), 7.12–7.27 (m, 5H), 7.02 (d, *J* = 8.5 Hz, 2H), 6.97 (d, *J* = 8.5 Hz, 1H), 6.77 (d, *J* = 15.7 Hz, 1H), 6.68 (d, *J* = 8.5 Hz, 2H), 5.52 (d, *J* = 5.8 Hz, 1H), 5.19 (d, *J* = 3.0 Hz, 1H), 5.14 (d, *J* = 4.3 Hz, 1H), 5.11 (d, *J* = 5.0 Hz, 1H), 5.05 (d, *J* = 5.1 Hz, 1H), 5.00 (s, 1H), 4.80 (d, *J* = 5.6 Hz, 1H), 4.74 (m, 1H), 4.67 (dd, *J* = 4.8, 9.2 Hz, 1H), 4.42 (m, 1H), 4.32–4.36 (m, 3H), 4.23 (s, 1H), 4.17 (dd, *J* = 7.2, 4.4 Hz, 1H), 3.94–4.03 (m, 5H), 3.87 (s, 3H), 3.83 (s, 3H), 3.79 (s, 3H), 3.78 (s, 3H), 3.67–3.90 (m, 4H), 3.20 (t, *J* = 9.4 Hz, 1H), 2.34–2.37 (m, 1H), 2.19–2.24 (m, 1H), 1.83–1.95 (m, 2H), 1.65–1.75 (m, 1H), 1.10 (m, 6H), 0.96 (d, *J* = 6.8 Hz, 3H). ^13^C NMR (125 MHz, DMSO-*d*_6_) δ 171.5, 170.7, 170.5, 169.7, 169.1, 168.2, 164.6, 156.6, 152.9, 140.9, 147.1, 138.7, 132.5, 131.1, 131.0, 130.4, 130.0, 129.0, 128.1, 121.7, 119.8, 117.5, 115.1, 114.7, 111.9, 109.8, 109.6, 75.4, 73.2, 73.1, 72.7, 69.3, 69.2, 68.3, 67.9, 66.2, 60.8, 60.6, 56.7, 55.7, 55.5, 55.3, 54.4, 51.2, 49.8, 37.3, 37.2, 34.7, 19.4, 19.2, 18.8, 18.0, 10.9.

### Biological Assays

#### Preparation of Stock Solutions of the Tested Compounds

PA was dissolved in DMSO in a 40 mg/mL stock solution. Compounds 2, 3, and 4 were dissolved in DMSO to a 20 mg/mL stock solution. Compound 5 was dissolved in DMSO in a 10 mg/mL stock solution. Echinocandin drugs and their derivatives, FLC, and propidium iodide, were dissolved in DMSO to 5 mg/mL stock solutions.

#### Fungal Strains

The laboratory and ATCC strains used in this study are listed in Table S1.

#### Growth Curve Analysis

Starter cultures were streaked from glycerol stock onto YPAD agar plates and grown for 24 h at 30 °C. Colonies were suspended in 1 mL of PBS and diluted to 1 × 10^–4^ optical density (OD_600_) in fresh medium. Stock solutions were added to growth media, and serial double dilutions were prepared in flat-bottomed 96-well microplates (Corning) to enable testing of concentrations ranging from 512 to 16 μg/mL. YPD was used as growth media. For assays of metals or polysaccharides effect, either metal or polysaccharide in the respective concentration (50 or 500 μg/mL of polysaccharides, and 10/100/1000 μM of metals) was added to the YPD growth medium. Control wells with yeast cells but no-drug (100% growth) and blank wells containing only growth medium (0% growth) were prepared. An equal volume (100 μL) of yeast suspensions in growth medium was added to each well except the blank wells. Growth was determined at 35 °C by measuring the OD_600_ using a plate reader (SPARK, Tecan, equipped with Spark-Stack) every 40 min. Each concentration was tested in triplicate, and the results were confirmed by at least two independent sets of experiments.

#### Disk Diffusion Assay

Fungal strains were streaked from glycerol stocks onto YPAD agar plates and grown for 24 h at 30 °C. Two or three colonies were placed into 1 mL of PBS solution, and OD_600_ was adjusted to 0.0005 for *Candida* strains and 0.005 for *S. cerevisiae* strain by dilution with PBS. Aliquots of 200 μL of the diluted cultures of each strain were plated onto 15 mL YPD agar plates and spread using sterile beads (3 mm, Fischer Scientific). After the plates dried, a single disk (6 mm diameter, Becton Dickinson) with 25 μg of tested compound was placed in the center of each plate. Plates were then incubated at 30 °C and photographed under the same imaging conditions after 24 h. The cell-wall-targeting caspofungin was used as a control drug.

#### Minimal Inhibitory Concentration (MIC) Broth Double-Dilution Assay

MIC values were determined by using CLSI M27-A3 guidelines with minor modifications. Starter cultures were streaked from glycerol stock onto YPAD agar plates and grown for 24 h at 30 °C. Colonies were suspended in 1 mL of PBS and diluted to 0.01 optical density (OD_600_) into fresh medium. Echinocandins and derivative stock solutions were added to YPD broth, and serial double dilutions of compounds in YPD were prepared in flat-bottomed 96-well microplates (Corning) to enable testing of concentrations ranging from 64 to 0.125 μg/mL for derivatives and from 1 to 3.9 × 10^–3^ μg/mL for parent drugs. Control wells with yeast cells but no-drug and blank wells containing only YPD were prepared. An equal volume (100 μL) of yeast suspensions in YPD broth was added to each well with the exception of the blank wells. MIC values (Table S2) were determined after 24 h at 30 °C by measuring the OD_600_ using a plate reader (Infinite M200 PRO, Tecan). MIC values were defined as the point at which the OD_600_ was reduced by ≥80% compared with the no-drug wells. Each concentration was tested in triplicate, and results were confirmed by two independent sets of experiments. Caspofungin was used as control drugs.

#### Live Cell Imaging

*S. cerevisiae* strain BY4741 was streaked from glycerol stocks onto YPAD agar plates and grown for 24 h at 30 °C. Colonies were then grown in 5 mL of YPD broth for 24 h at 30 °C with shaking in tubes. Cultures were diluted 1:50 and incubated in YPD broth for 3 h at 30 °C with shaking until log-phase growth was observed. PA (final concentration 1 mM) or an equal volume of DMSO was added, and the cultures were incubated with shaking at 30 °C for 1, 2, 3, 24, and 42 h. At each time point, 1 mL of culture was pelleted. The YPD was removed, and the pellet was resuspended in 1 mL of PBS. Cells were stained with CFW (25 μL, final concentration 25 μg/mL) for 5 min at room temperature. After staining, the cultures were centrifuged, washed with 1 mL of PBS, and pelleted. Pellets were resuspended in PBS according to pellet size, and a 2 μL aliquot of cell sample was placed on a glass slide and covered with a glass coverslip. Cells were imaged on a Nikon Ti2 microscope equipped with a Plan Apo λ 100× Oil objective and a Prime BSI A21H204007 camera using NIS elements Ar software. The bandpass filter set used to image CFW had an excitation wavelength of 377/50 nm and an emission wavelength of 447/60 nm. Images were processed using ImageJ software.

#### Flow Cytometry

*S. cerevisiae* strain was streaked from glycerol stocks onto YPAD agar plates and grown for 24 h at 30 °C. Colonies were then grown in 5 mL of YPD broth for 24 h at 30 °C with shaking in tubes. Cultures were diluted at 1:50 and incubated in YPD broth for 3 h at 30 °C with shaking until log-phase growth was observed. PA (final 1 mM), caspofungin (final 1 μM), or DMSO were added from stock solutions and incubated with shaking at 30 °C. At each time point (10 min, 3, 6, 22, 28 h) 200 μL samples were pelleted, YPD was removed, pellets were washed twice with 200 μL of PBS and then resuspended in 200 μL of PBS. For chitin content assay, 5 μL of CFW was added (final concentration 25 μg/mL) and incubated for 5 min at 37 °C with shaking. For permeability assay, 1 μL of propidium iodide (PI) was added (final concentration 5 μg/mL) and incubated for 15 min at 37 °C with shaking. Samples were transferred to flat-bottomed 96-well microplates (Corning) and read by MACSQuant VYB flow cytometer. For PI assay, a 614/50 nm filter was used. For CFW assay, a 450/50 nm filter was used.

## Supporting Information Available

Yeast strains (Table S1); growth curves (Figures S1–S3); chitin–PA interaction analysis (Figure S4); live cell imaging data (Figure S5); disk diffusion assay (Figure S6); detailed synthetic schemes (Schemes S1–S2); MIC values (Table S2); analytic HPLC chromatograms (Figures S7–S16); and ^1^H and ^13^C NMR spectra (Figures S17–S34) (PDF)

## Abbreviations

**PA**: poacic acid
**DNA**: deoxyribonucleic acid
**CYP51**: cytochrome P450
**OD**_**600**_: optical density (600 nm)
**PS**: phosphatidylserine
**YPD**: yeast extract peptone dextrose medium
**YPAD**: yeast extract peptone dextrose medium plus adenine
**PBS**: phosphate-buffered saline
**CFW**: calcofluor white
**PI**: propidium iodide
**DMSO**: dimethyl sulfoxide
**DIC**: differential interference contrast
**log *D***: the distribution coefficient
**MIC**: minimal inhibitory concentration
**DMF**: dimethyl formamide
**THF**: tetrahydrofurane
**HOBt**: hydroxybenzotriazole
**DCC**: *N*,*N*′-dicyclohexylcarbodiimide
**DIPEA**: *N*,*N*-diisopropylethylamine
**DCM**: dichloromethane
**HRMS**: high-resolution mass spectroscopy
**RP-HPLC**: reversed-phase high-performance liquid chromatography
**ddH**_**2**_**O**: double-distilled water
**TLC**: thin-layer chromatography
**TFA**: trifluoro acetic acid
**NHS**: *N*-hydroxysuccinimide
**TEA**: triethylamine
**ATCC**: the American Type Culture Collection
