# Supplementary material for: Poacic Acid, a Plant-Derived Stilbenoid, Augments Cell Wall Chitin Production, but Its Antifungal Activity Is Hindered by This Polysaccharide and by Fungal Essential Metals
Source: Biochemistry. 2024 Mar 27;63(8):1051–65. doi: 10.1021/acs.biochem.3c00595 (PMC11025111; doi:10.1021/acs.biochem.3c00595)
Supplement: Supplementary file 1 — bi3c00595_si_001.pdf [file bi3c00595_si_001.pdf]

Supporting Information

Poacic Acid, a Plant-Derived Stilbenoid, Augments Cell-Wall Chitin Production, but Its Antifungal Activity Is Hindered by this Polysaccharide and by Fungal Essential Metals

Adi Yona, [a] *and* Micha *Fridman* [a,*]

[a] School of Chemistry, Raymond & Beverly Sackler Faculty of Exact Sciences, Tel Aviv University, Tel Aviv, 6997801, Israel.

*Correspondence to: Micha Fridman, mfridman@tauex.tau.ac.il

Table of Contents

1. Biological Assessment of PA and Its Derivatives

1.1. Yeast strains 2

1.2. Growth curves analysis 2

1.3. Chitin interaction analysis 4

1.4. Live cell imaging 5

1.5. Minimal inhibitory concentration assays 6

1.6. Disc diffusion assay 7

2. Synthesis and Compound Characterization Information

2.1. Synthetic schemes 8

2.2. Analytical HPLC chromatograms 9

2.3. ^{1}H-NMR spectra 14

2.4. ^{13}C-NMR spectra 18

3. References 23

1. Biological Assessment of PA and Its Derivatives

1.1. Yeast strains

Table S1. Strain information.

#	Species	Strain name	Isogenic parental strain	Genotype	Source
**A.**	*C. albicans*	SC5314	WT		David Perlin[1]
**B.**	*C. albicans*	SN152	-		Susan Lindquist[2]
**C.**	*C. albicans*	YLC337	SC5314	cho1Δ/Δ	Todd B. Reynolds[3]
**D.**	*C. albicans*	BV11	SN152	erg3Δ/Δerg11Δ/Δ	Susan Lindquist[2]
**E.**	*C. glabrata*	ATCC 66032	-		ATCC
**F.**	*C. parapsilosis*	ATCC 22019	-		ATCC
**G.**	*S. cerevisiae*	BY4741	WT		Jef D. Boeke[4]
**H.**	*S. cerevisiae*	BY4743	-		Jef D. Boeke[4]

1.2. Growth curves analysis

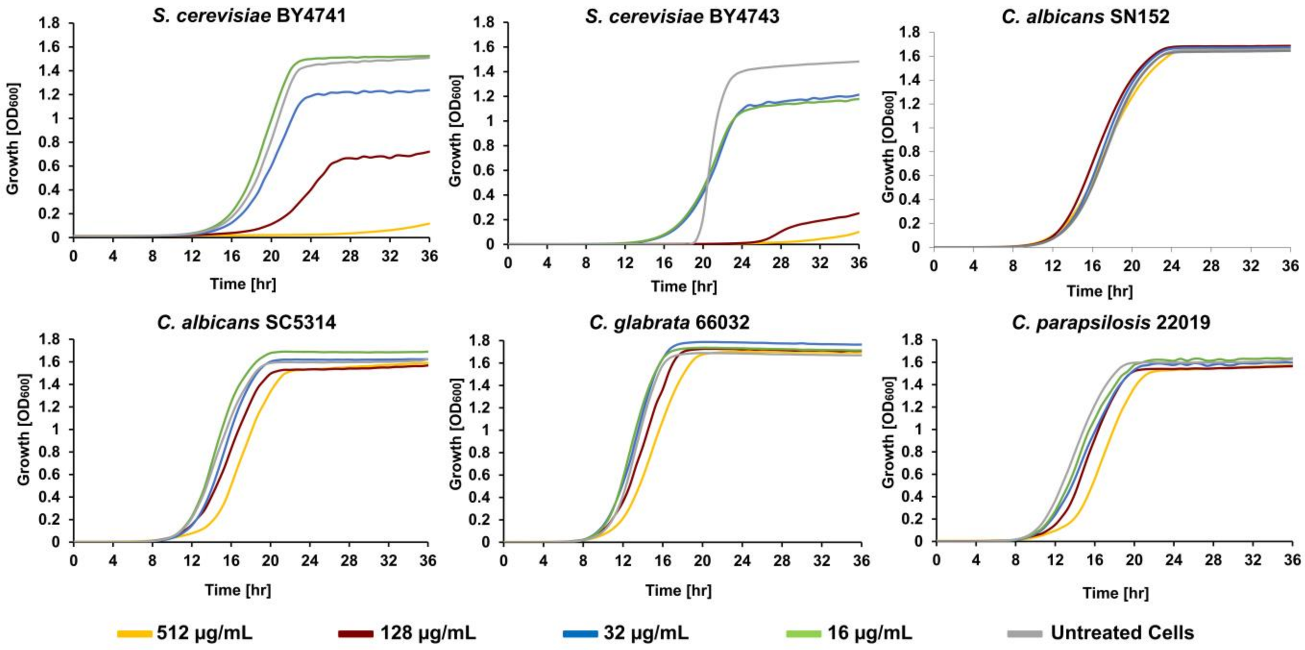

Figure S1. Growth curves of fungi strains treated with varying concentrations of PA.

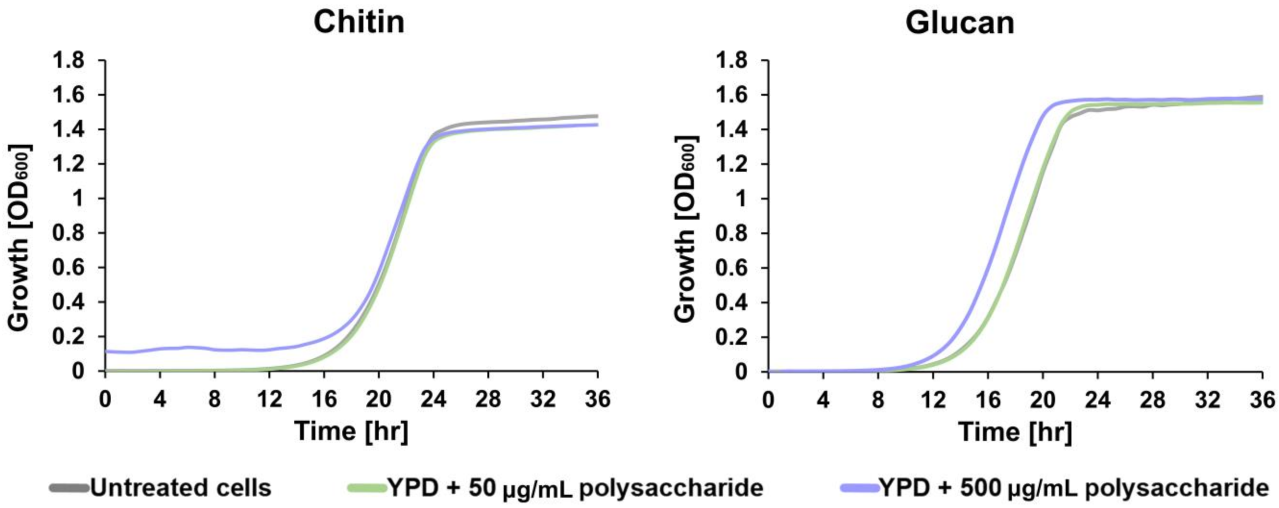

Figure S2. Control growth curves of BY4741 cells grown in YPD broth enriched with respective polysaccharide.

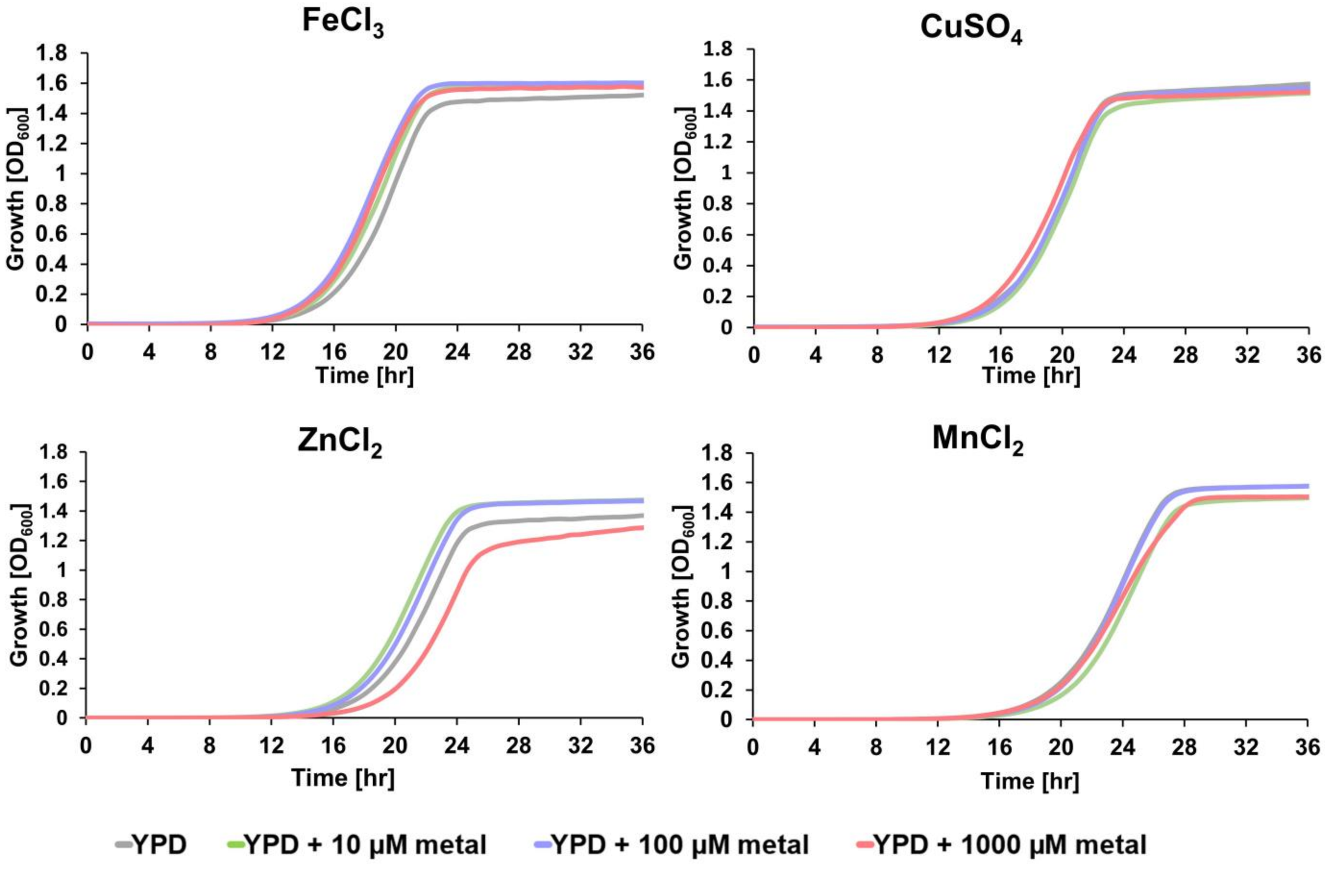

Figure S3. Control growth curves of BY4741 cells grown in YPD broth enriched with respective metal ions.

1.3. Chitin-PA complex

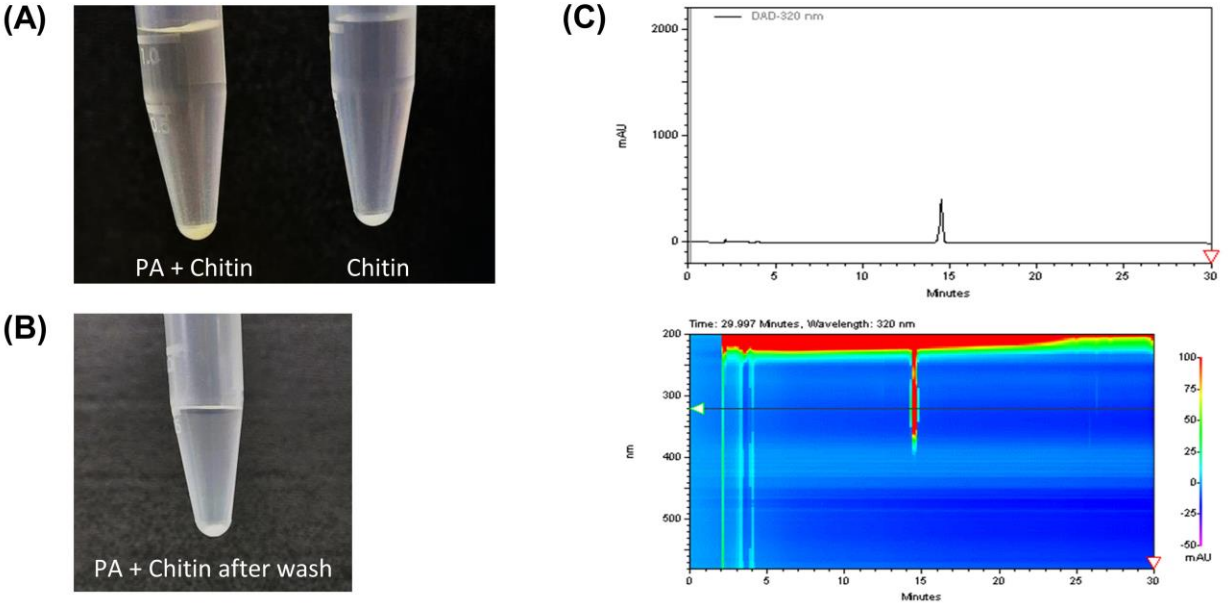

Figure S4. (A) Precipitate of chitin (1000 µg/mL) and PA (512 µg/mL) in PBS (1mL) is seen in light yellow (left), whereas precipitate of chitin (1000 µg/mL) without PA is white (right). **(B)** Supernatant from chitin + PA sample was removed, and precipitate washed with 1 mL PBS to remove remaining solubilized PA. The precipitate was then dissolved in 0.5 mL ACN:H_2O (8:2) solution. The color of precipitate returns to white indicating the unbinding of PA from chitin. **(C)** Analytical HPLC chromatograms of supernatant from B showing absorption and elution time consistent with PA (320 nm, 14.8 min). HPLC conditions: mobile phase: ACN/ H_2O (containing 0.1% TFA), gradient of 10% to 90% ACN; flow rate: 1 mL/min.

1.4. Live cell imaging

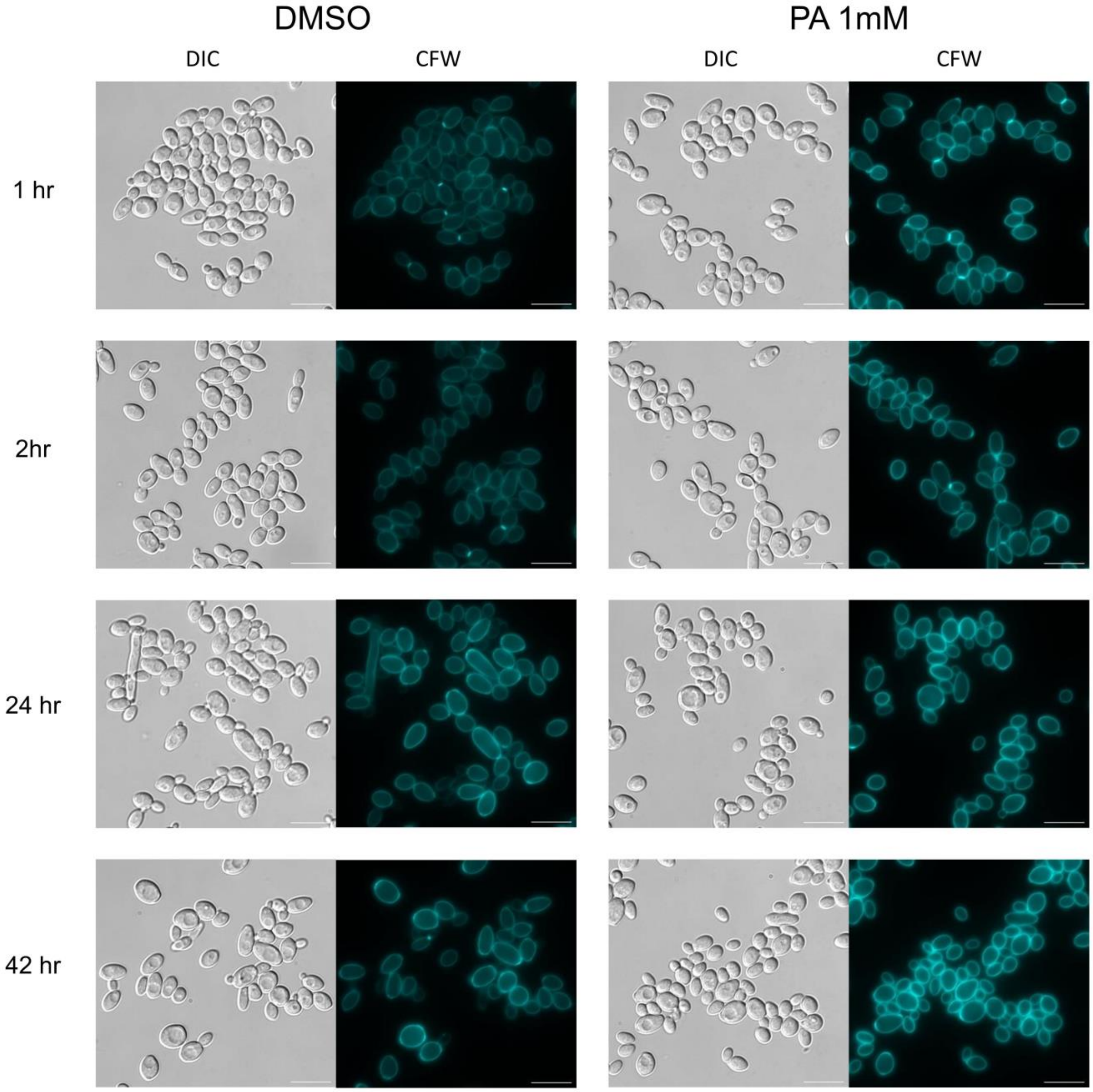

Figure S5. Time-dependent effect of PA on *C. albicans* strain SN152 cell wall chitin production and distribution as visualized by calcofluor white (CFW) stain. Differential interference contrast (DIC) and fluorescent images of cells incubated for one, two, 24 or 42 hours in YPD and either 1mM PA (right) or DMSO (left). Cells were washed and stained with CFW prior observation. Scale bars, 10 µm. A bandpass filter with an excitation of 377/50 nm and an emission wavelength of 447/60 nm was used for CFW. Similar images were obtained in at least two independent experiments.

1.5. Minimal inhibitory concentration assays

Table S2. Minimal inhibitory concentration (MIC) values of tested compounds.

			Compound MIC [μg/mL]				
	**Strain name**	**Media**	**CSF**	**6**	**7**	**8**	**9**
A.	SN152	YPD	0.008	2	4	>64	>64
		YPD + Chitin	0.016	4	nd	nd	nd
B.	BY4741	YPD	0.063	2	2	>64	>64
		YPD + Chitin	0.063	8	nd	nd	nd
C.	ATCC 66032	YPD	0.031	1	1	>64	>64
D.	ATCC 22019	YPD	0.125	2	2	>64	>64

All MICs were determined using the broth double-dilution method starting from a concentration of 64 μg/mL. Cells were grown in YPD at 30 °C for 24 h. Each concentration was tested in triplicate, and results were confirmed by two independent sets of experiments. Exogenous chitin was added to a final concentration of 500 μg/mL. MIC_{80} was determined as the minimal concentration at which the growth was lower than 20% compared to untreated cells.

[nd] MIC value was not determined.

1.6. Disc diffusion assay

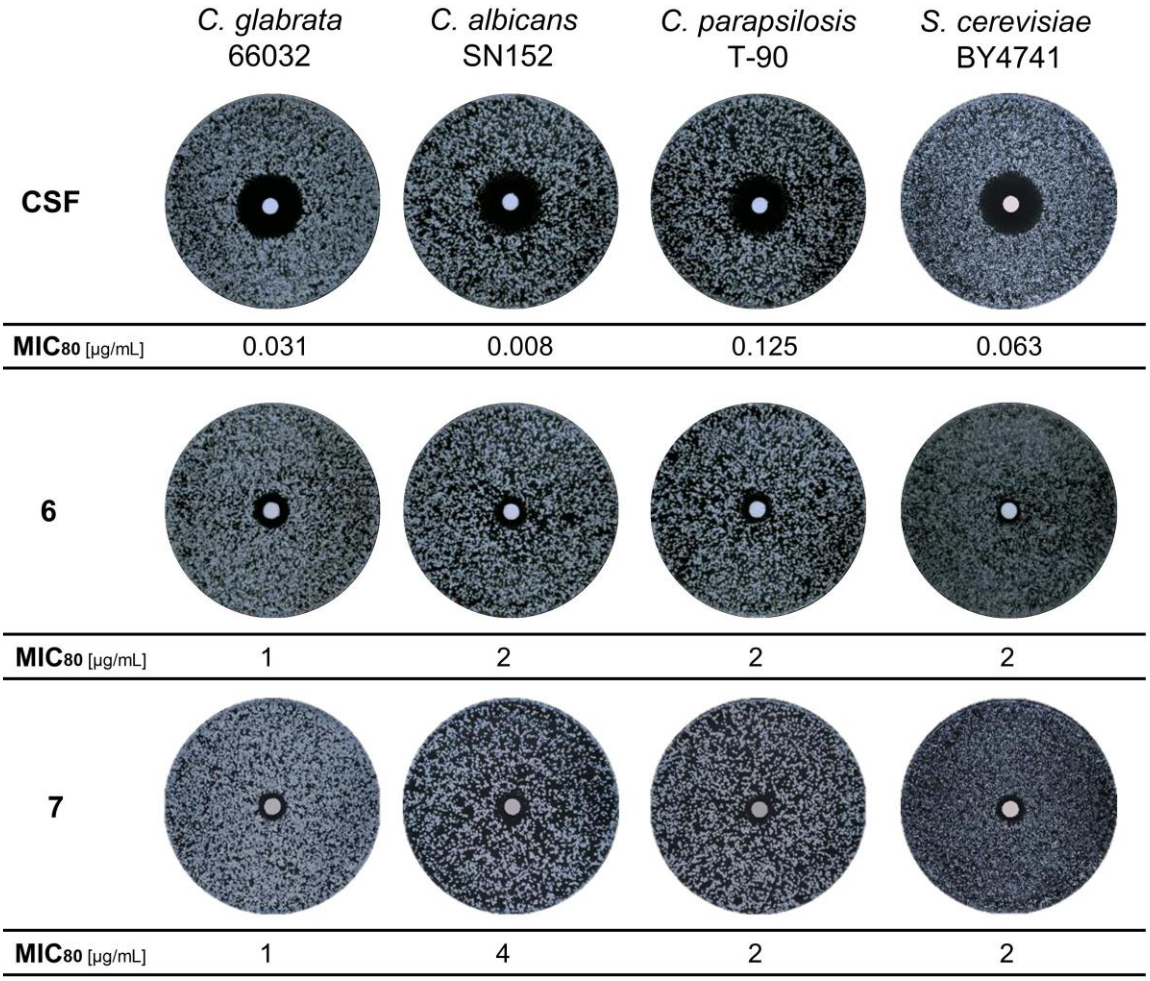

Figure S6. Disk diffusion assays of CSF and echinocandin-PA derivatives **6** and **7** on four fungal strains. Each YPD agar plate was loaded with 200 µL of fungi suspended in PBS to a final OD_{600} of 0.0005 for *Candida* strains, and 0.005 for *S. cerevisiae* strain. A sterile disk with 25 µg of tested compound was placed in the center the plates which were then incubated at 30°C for 24 hours.

2. Synthesis and Compound Characterization Information

2.1. Synthetic schemes

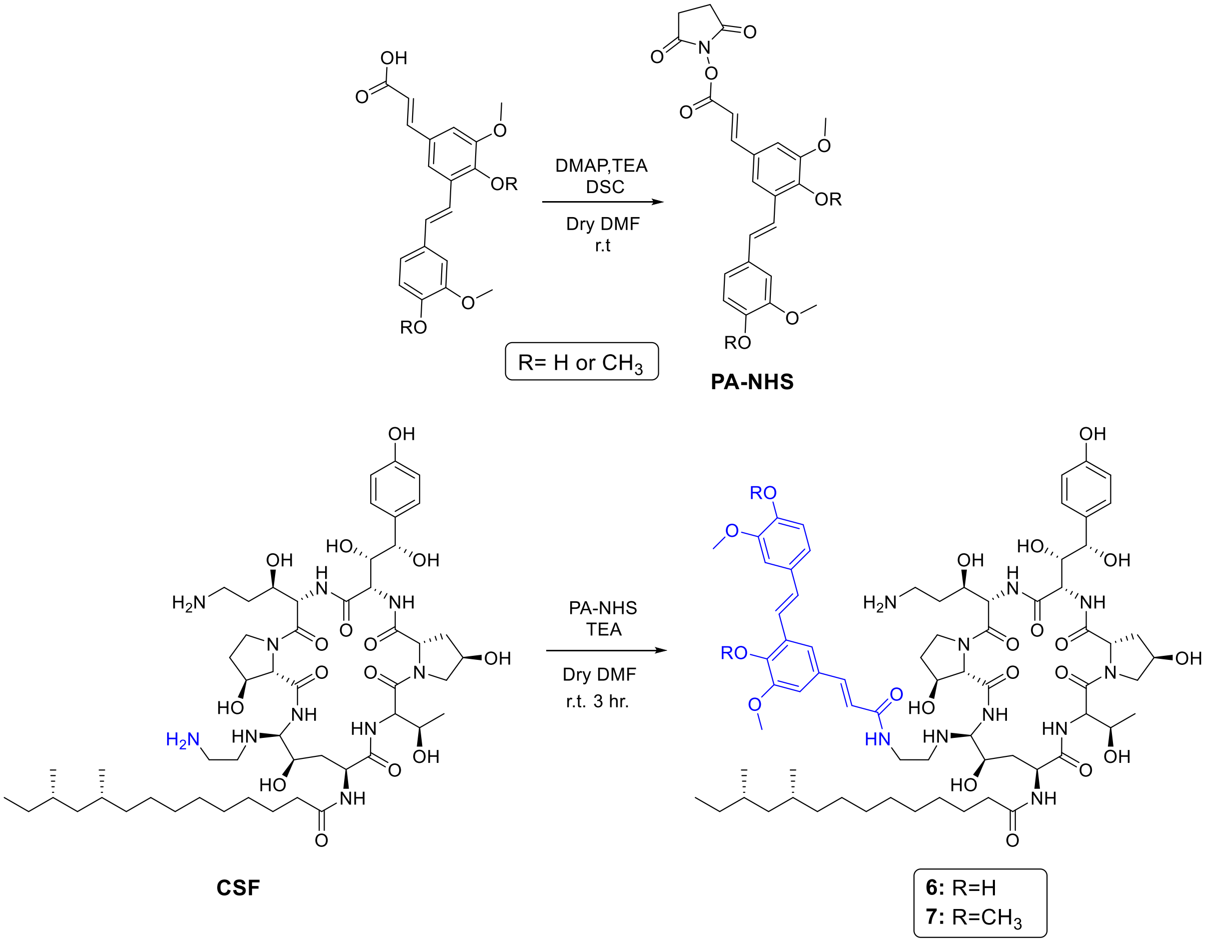

Scheme S1. Synthesis of the PA-functionalized caspofungin compounds **6** and **7**.

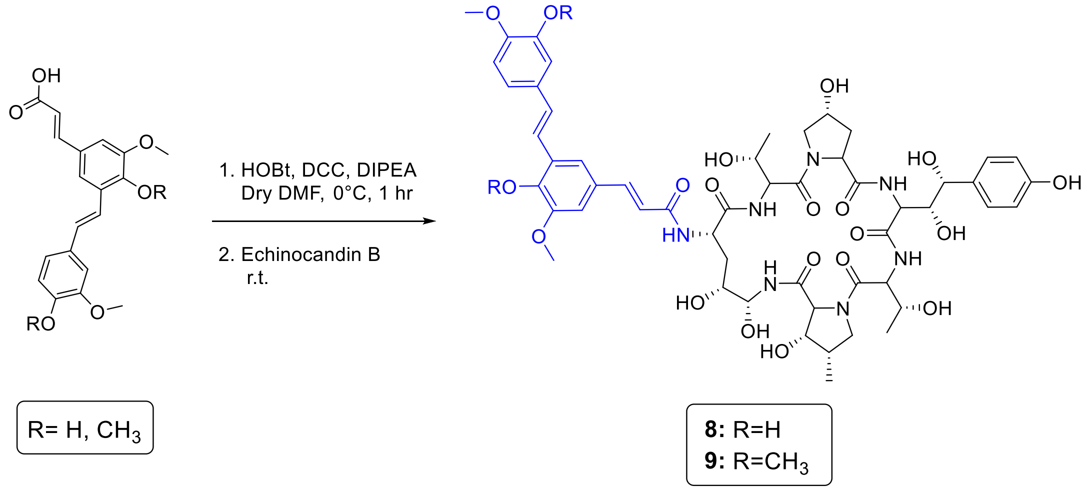

Scheme S2. Synthesis of the PA-functionalized echinocandin B compounds **8** and **9**.

2.2. Analytical HPLC chromatograms

Conditions: Alltech Apollo C18 reversed-phase column (5 μm, 4.6 x 250 mm); mobile phase: ACN/H_2O (containing 0.1% TFA), gradient from 10% to 90% ACN; flow rate: 1 mL/min.

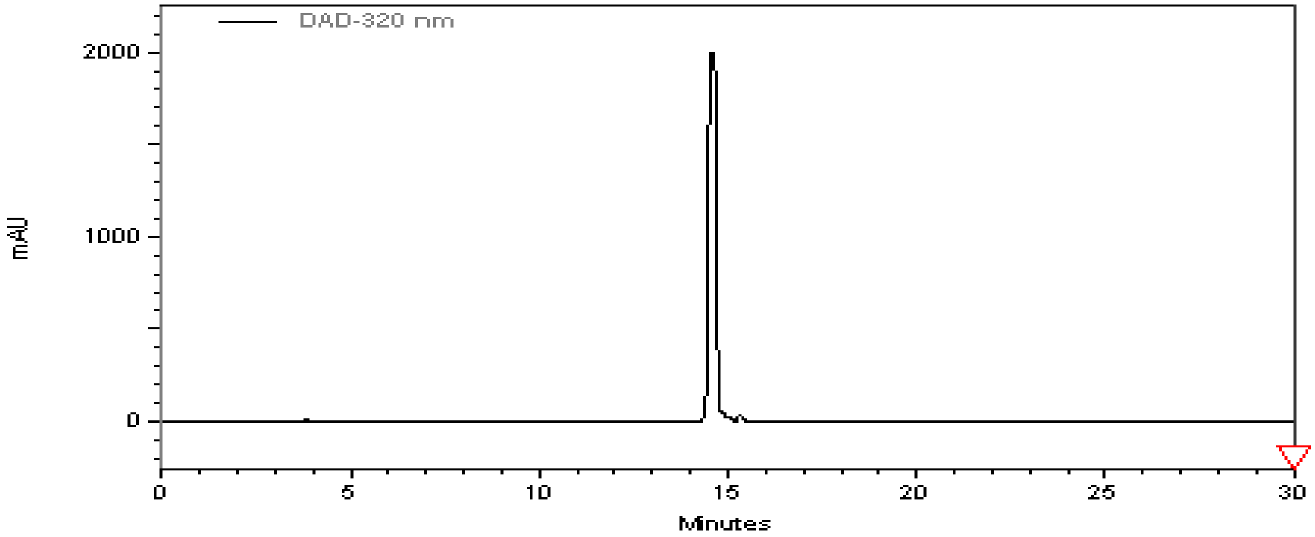

Figure S7. Analytical RP-HPLC chromatogram (diode array detector) of **PA**.

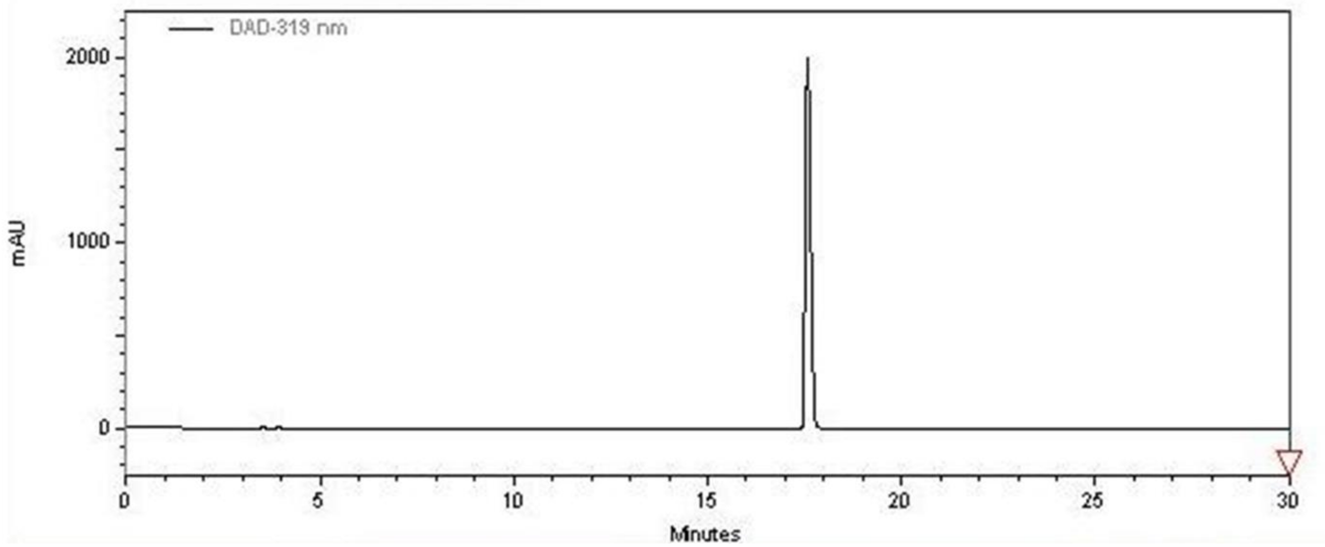

Figure S8. Analytical RP-HPLC chromatogram (diode array detector) of compound **1**.

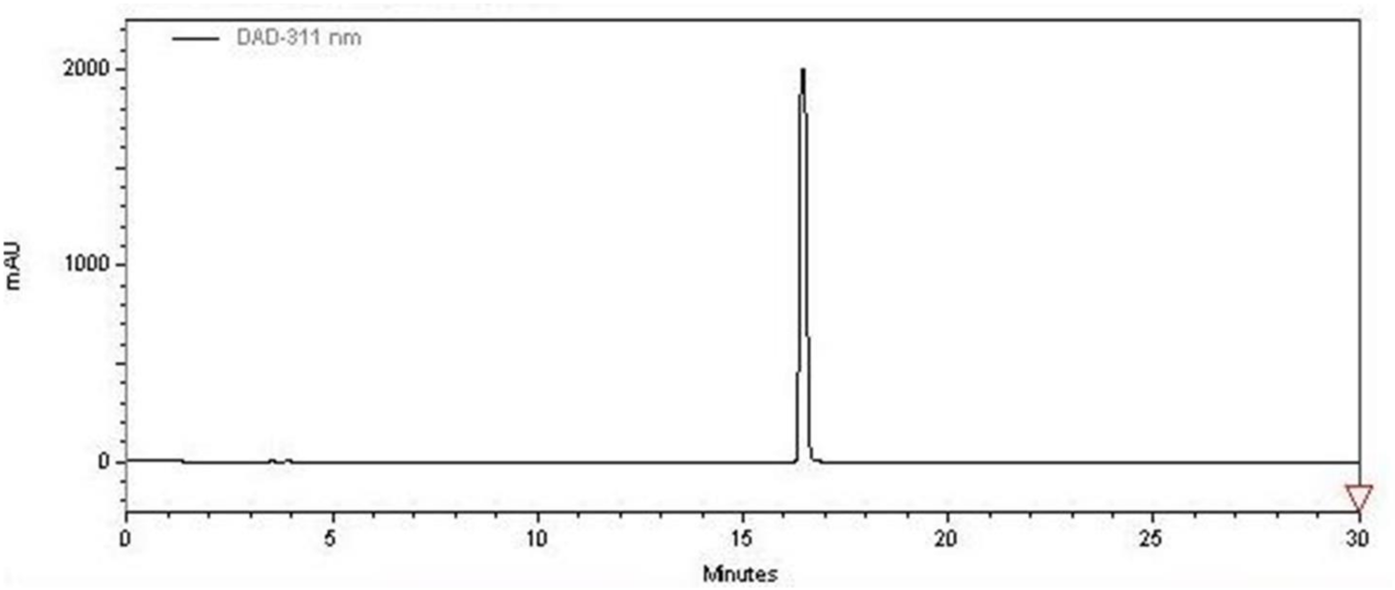

Figure S9. Analytical RP-HPLC chromatogram (diode array detector) of compound **2**.

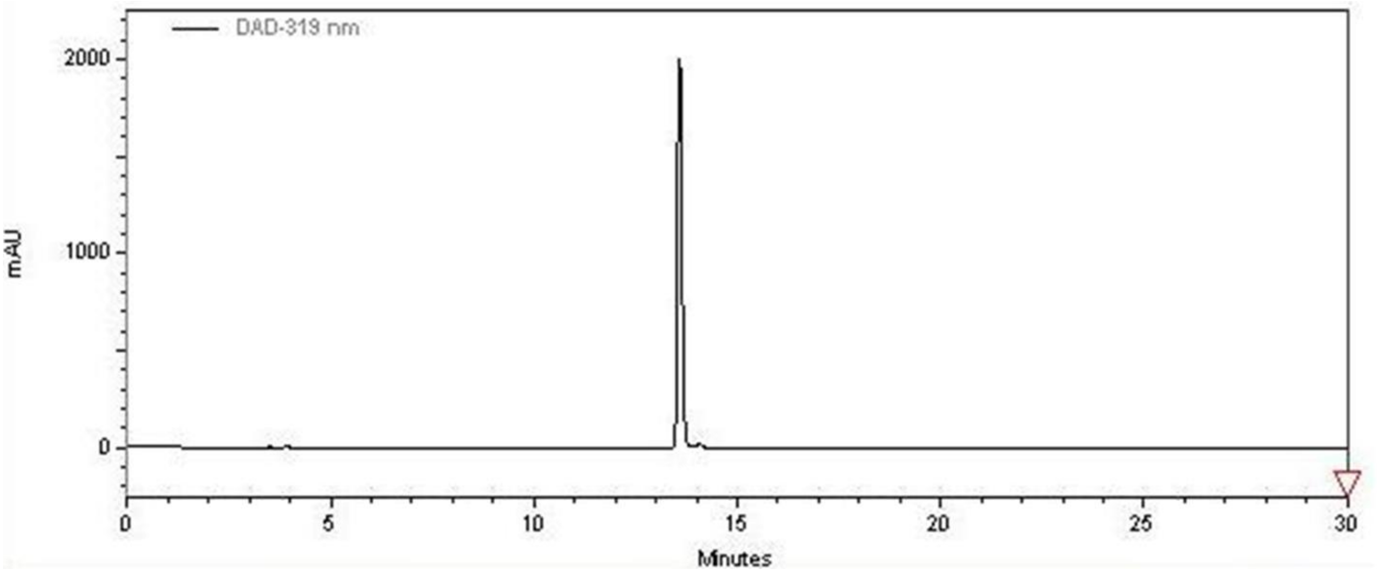

Figure S10. Analytical RP-HPLC chromatogram (diode array detector) of compound **3**.

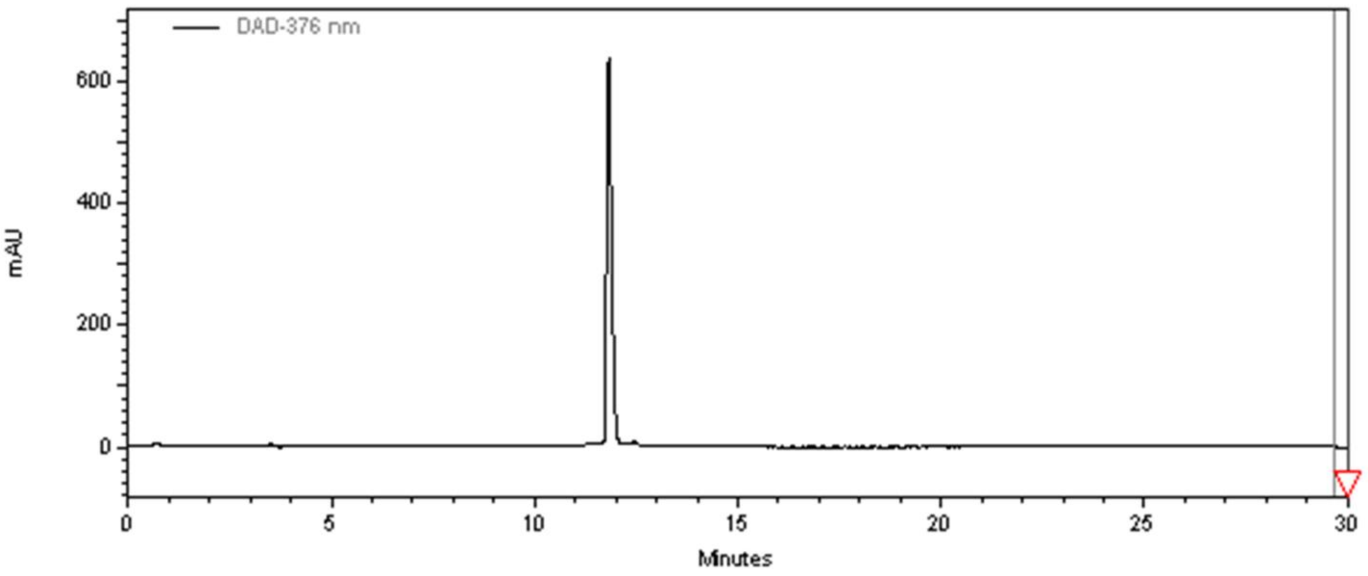

Figure S11. Analytical RP-HPLC chromatogram (diode array detector) of compound **4**.

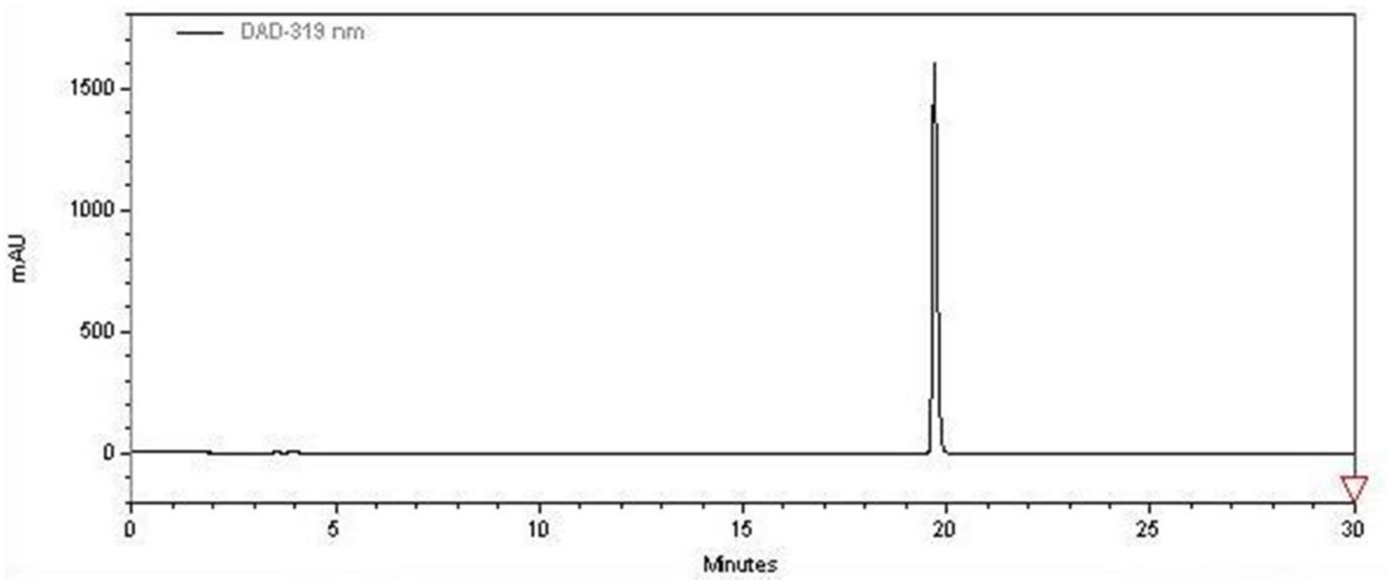

Figure S12. Analytical RP-HPLC chromatogram (diode array detector) of compound **5**.

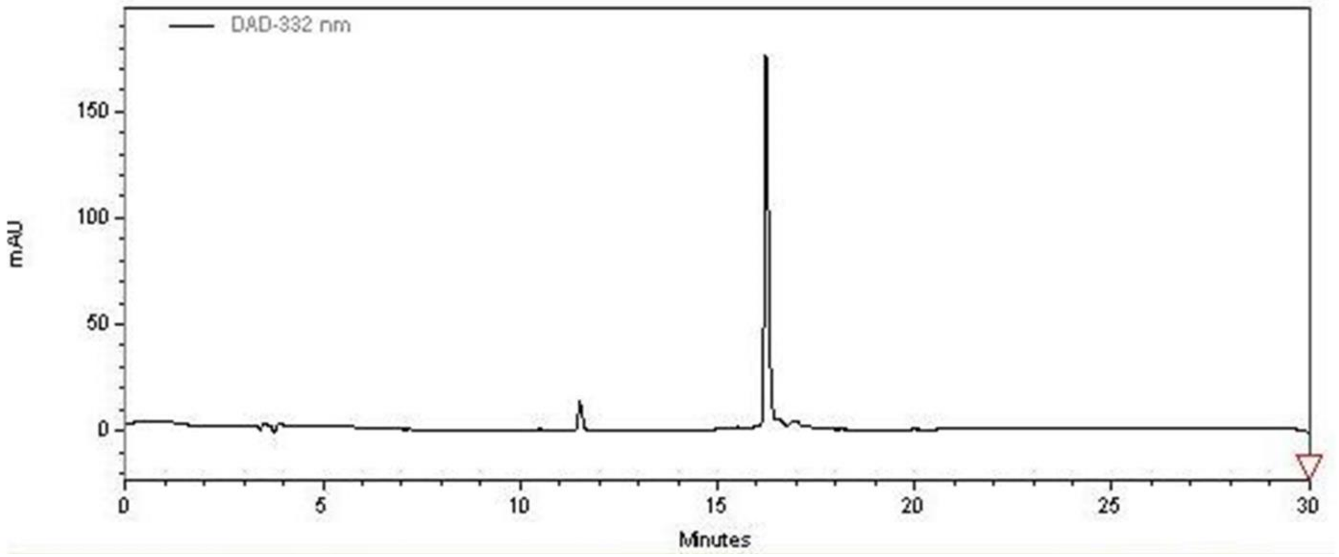

Figure S13. Analytical RP-HPLC chromatogram (diode array detector) of compound **6**.

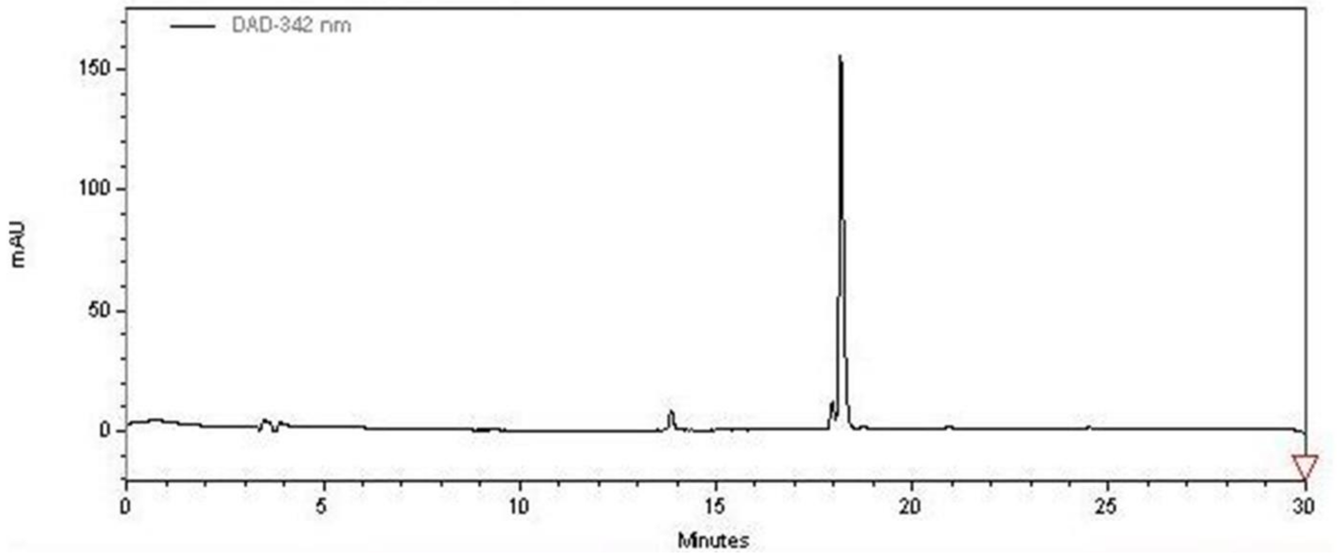

Figure S14. Analytical RP-HPLC chromatogram (diode array detector) of compound **7**.

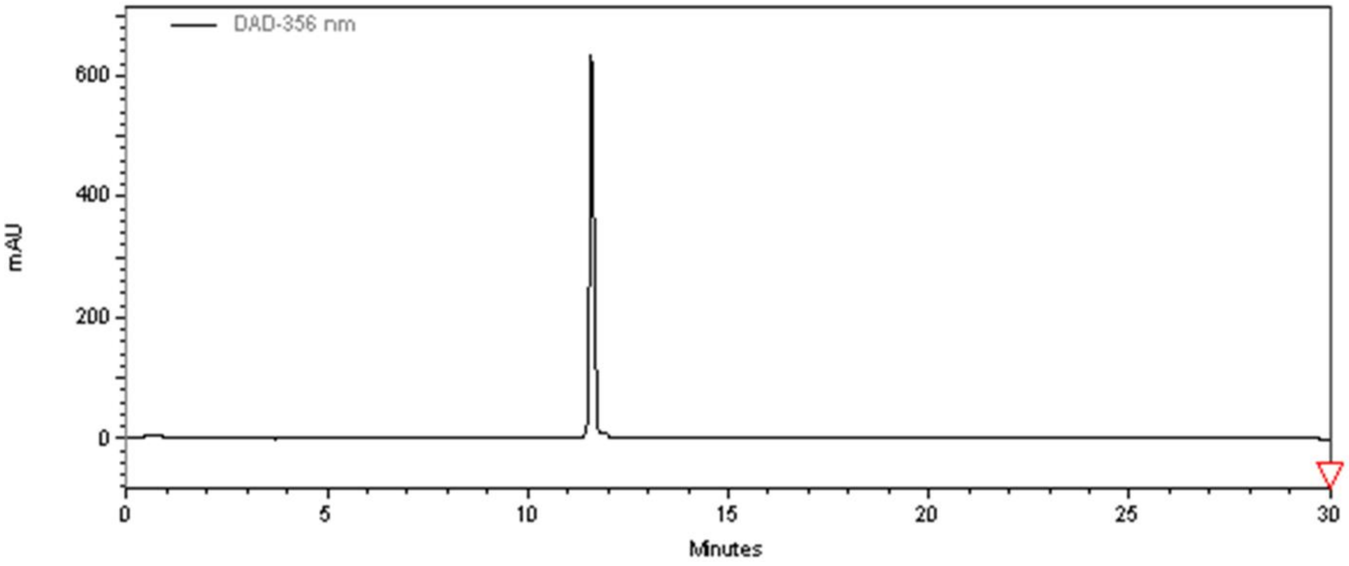

Figure S15. Analytical RP-HPLC chromatogram (diode array detector) of compound **8**.

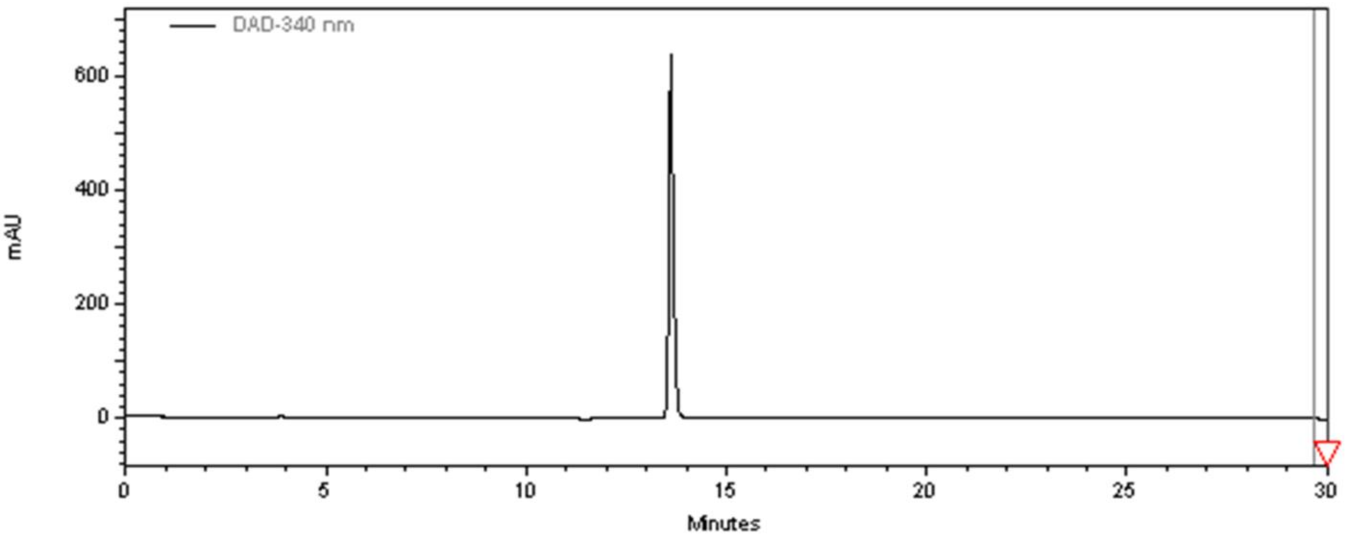

Figure S16. Analytical RP-HPLC chromatogram (diode array detector) of compound **9**.

2.3. ^{1}H-NMR spectra

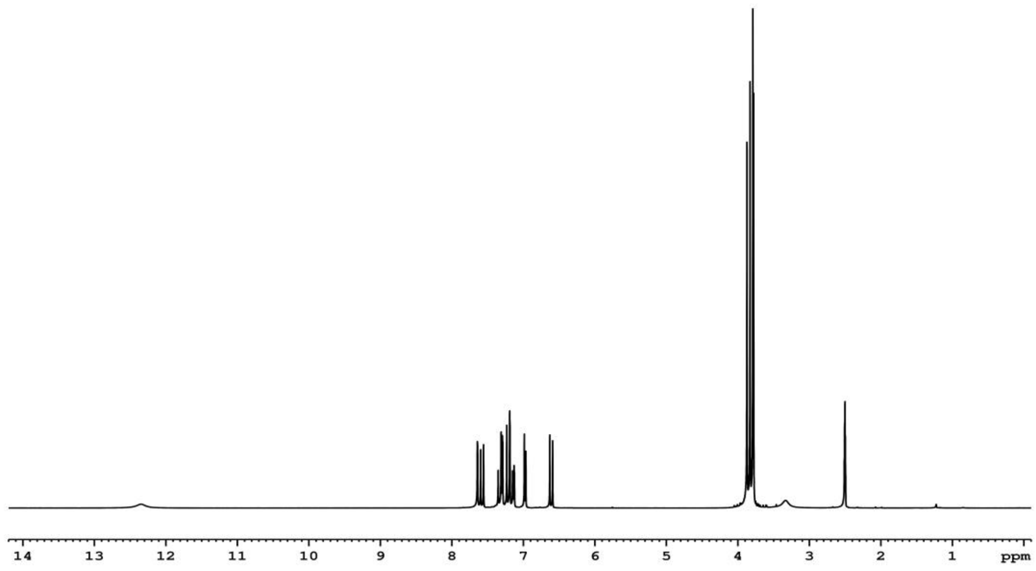

Figure S17. 400 MHz ^{1}H-NMR spectrum of compound **1** in DMSO-d_6.

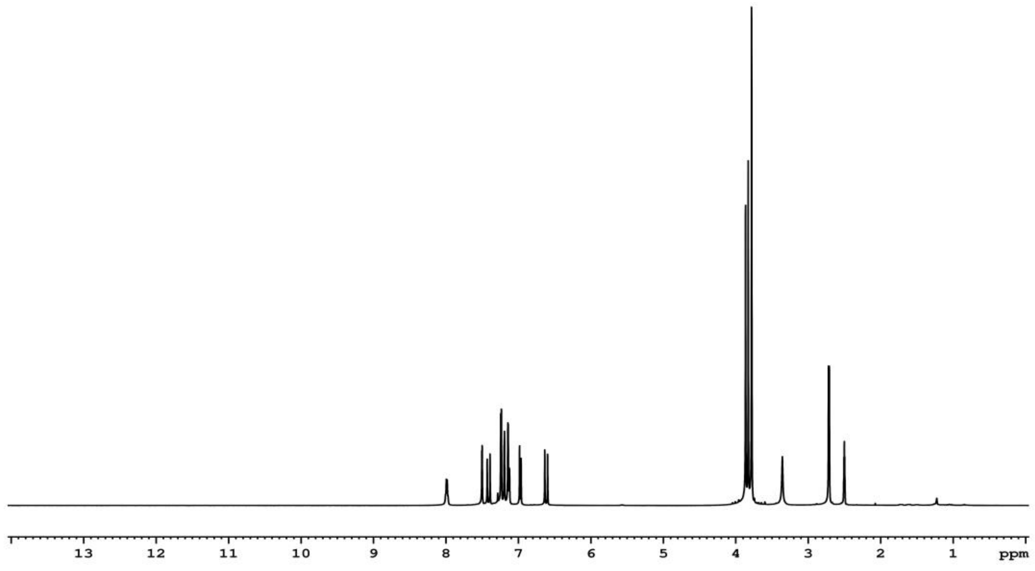

Figure S18. 400 MHz ^{1}H-NMR spectrum of compound **2** in DMSO-d_6.

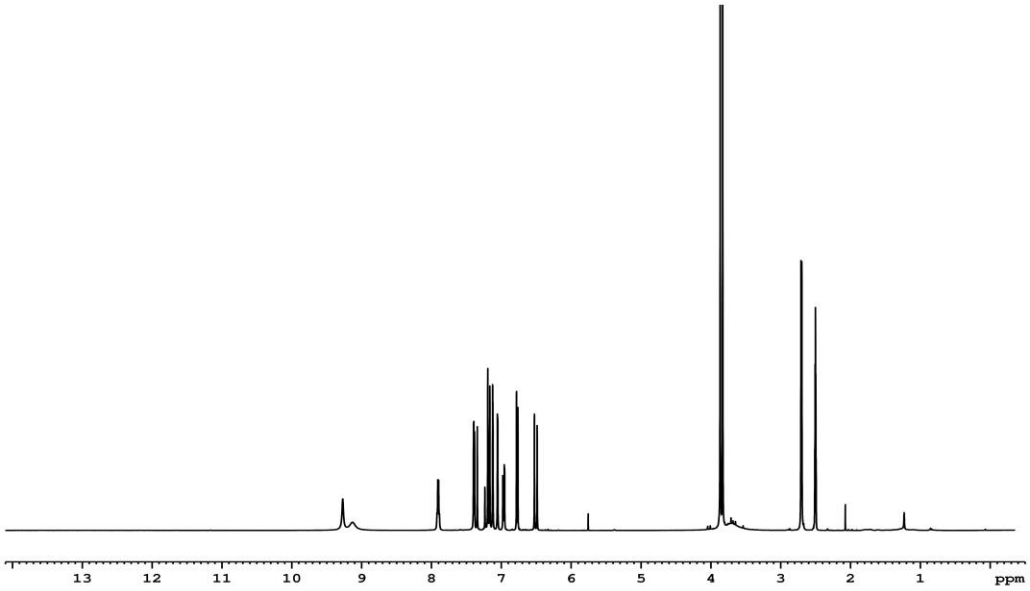

Figure S19. 400 MHz ^{1}H-NMR spectrum of compound **3** in DMSO-d_6.

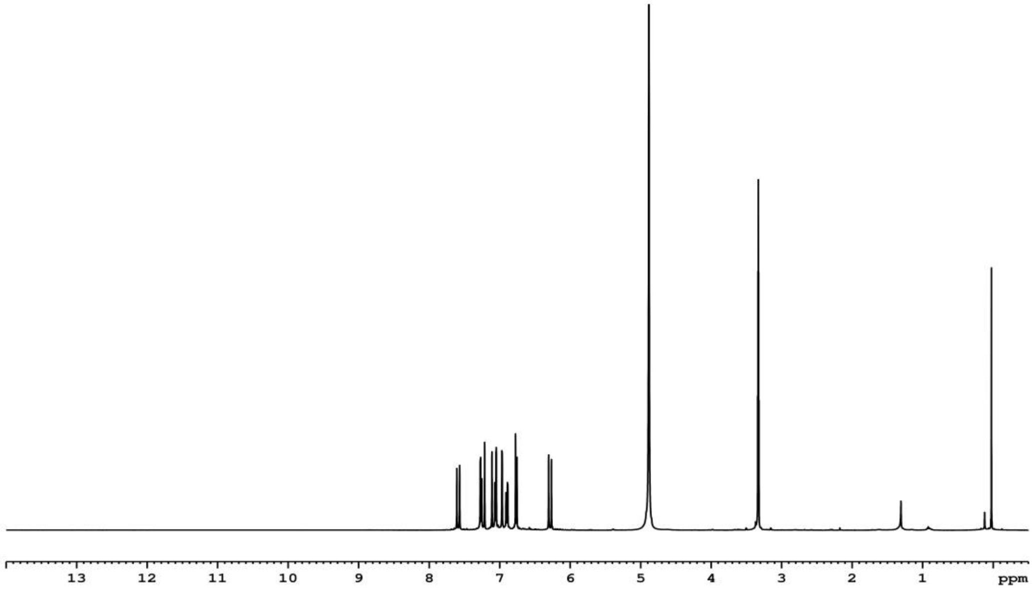

Figure S20. 400 MHz ^{1}H-NMR spectrum of compound **4** in DMSO-d_6.

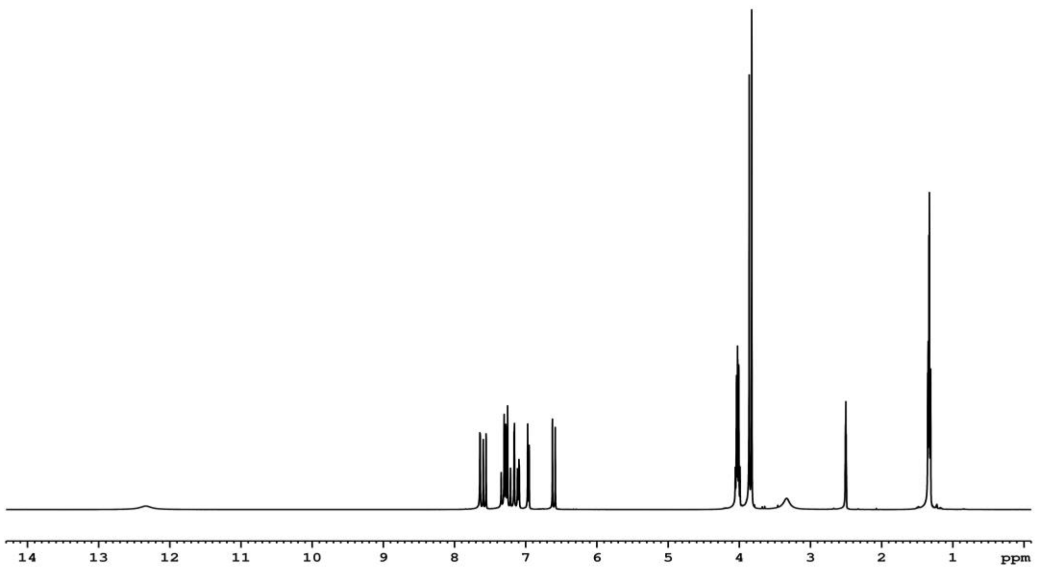

Figure S21. 400 MHz ^{1}H-NMR spectrum of compound **5** in DMSO-d_6.

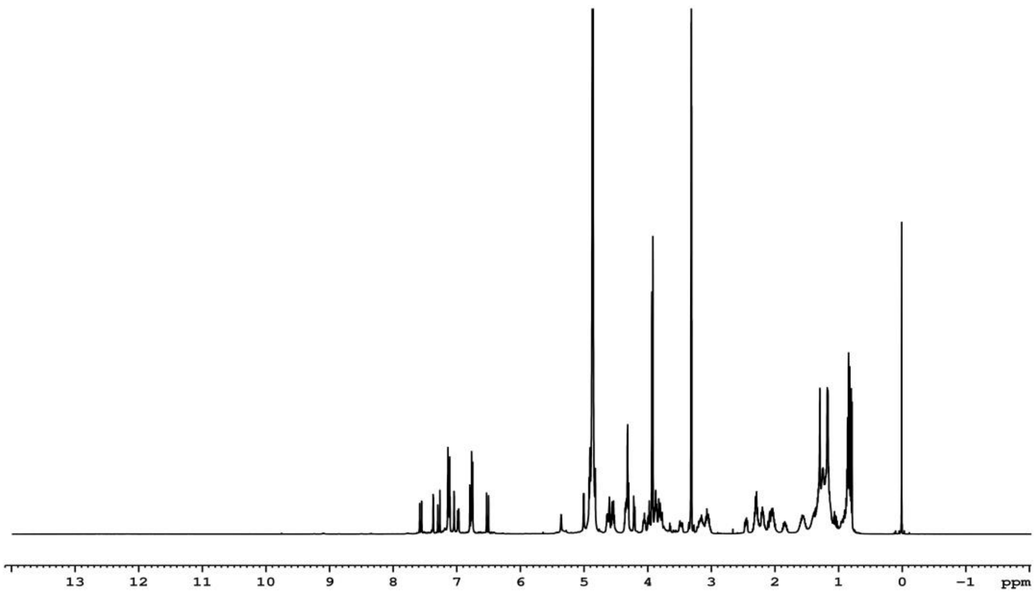

Figure S22. 500 MHz ^{1}H-NMR spectrum of compound **6** in CD_3OD.

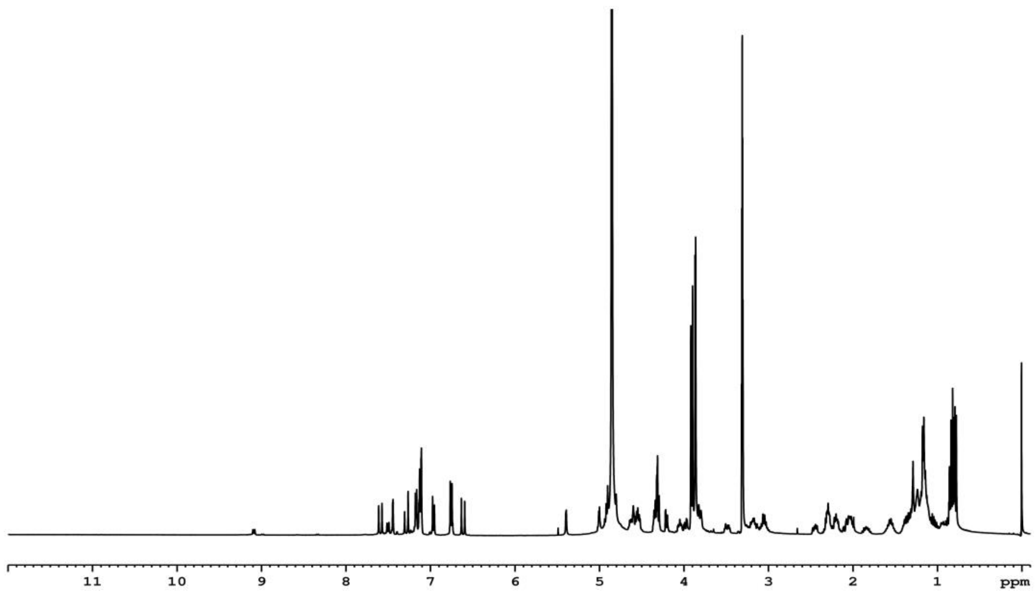

Figure S23. 500 MHz ^{1}H-NMR spectrum of compound **7** in CD_3OD.

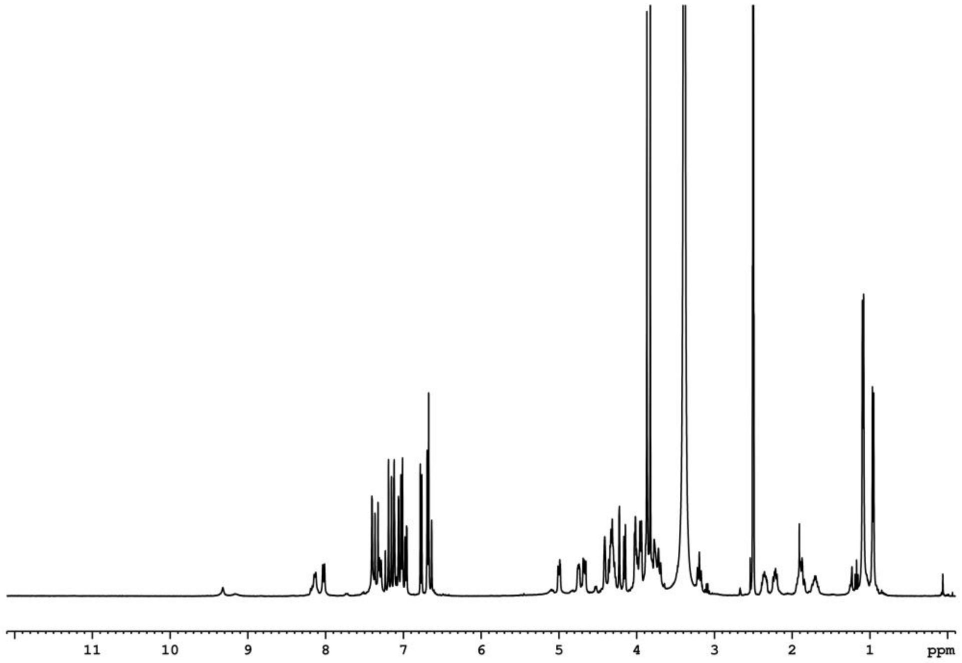

Figure S24. 400 MHz ^{1}H-NMR spectrum of compound **8** in DMSO-d_6.

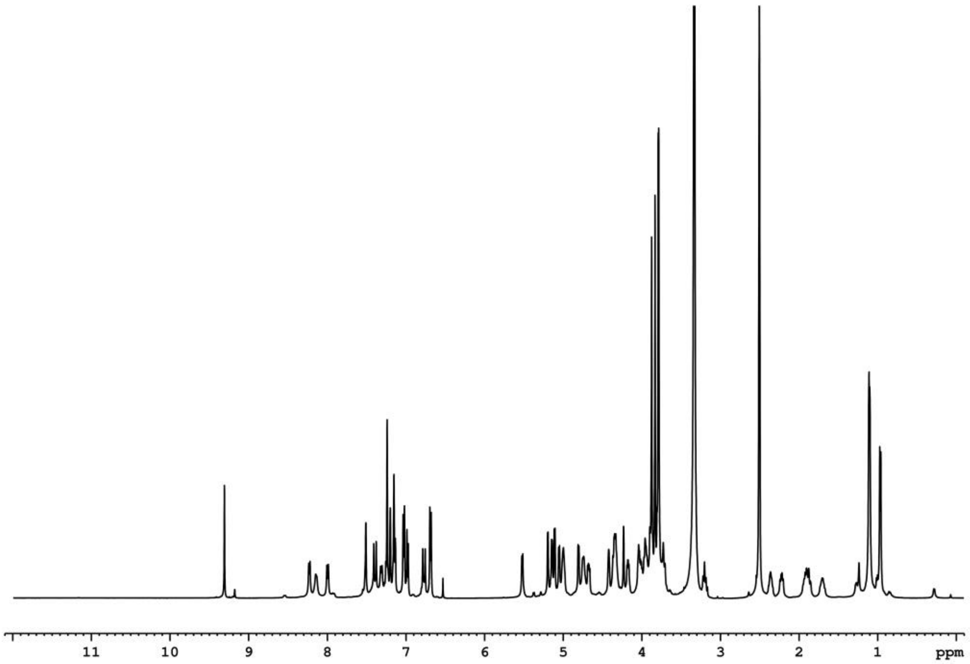

Figure S25. 500 MHz ^{1}H-NMR spectrum of compound **9** in DMSO-d_6.

2.4. ^{13}C-NMR spectra

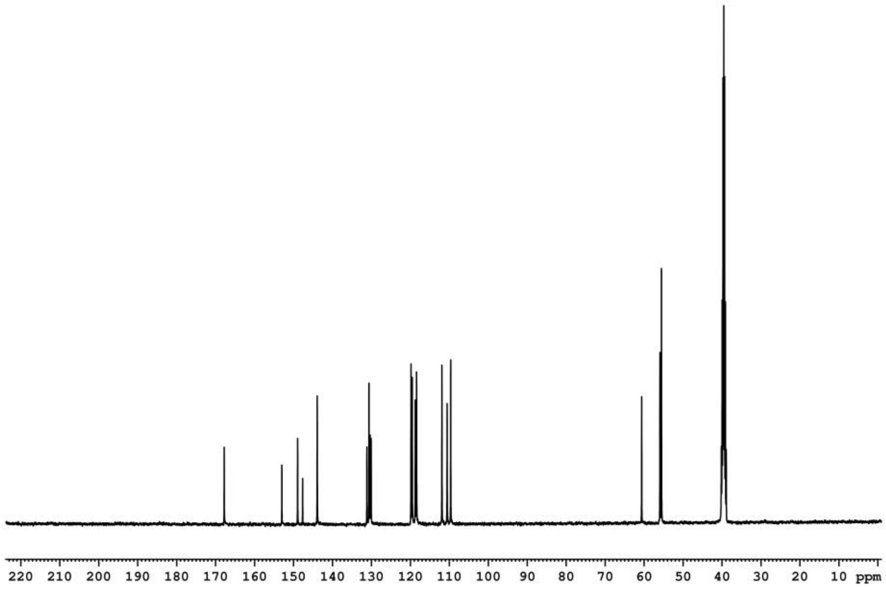

Figure S26. 100 MHz ^{13}C-NMR spectrum of compound **1** in DMSO-d_6.

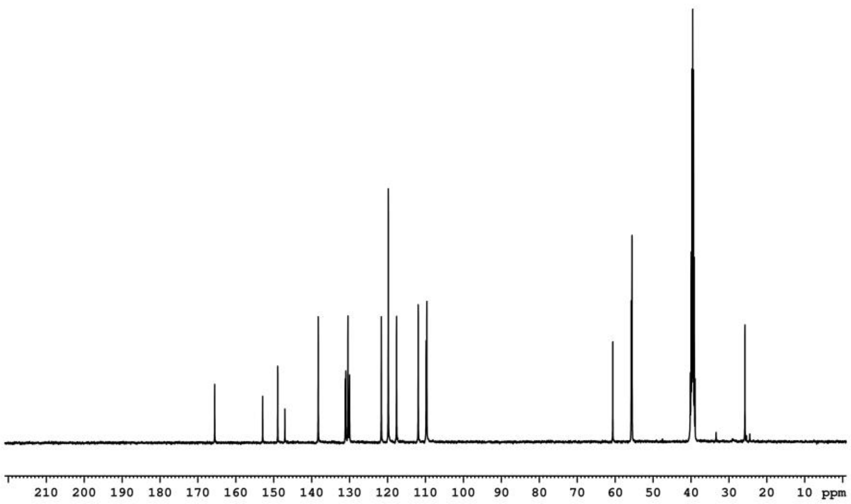

Figure S27. 100 MHz ^{13}C-NMR spectrum of compound **2** in DMSO-d_6.

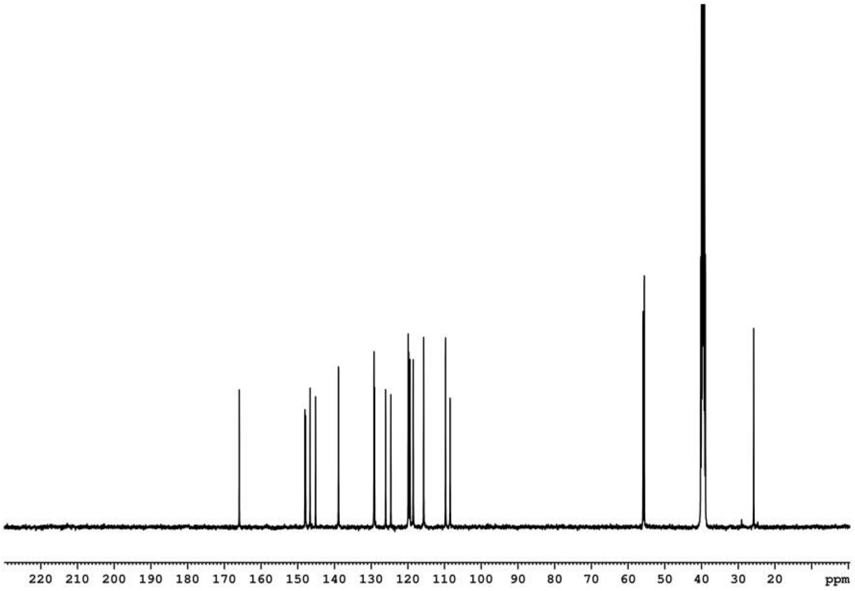

Figure S28. 100 MHz ^{13}C-NMR spectrum of compound **3** in DMSO-d_6.

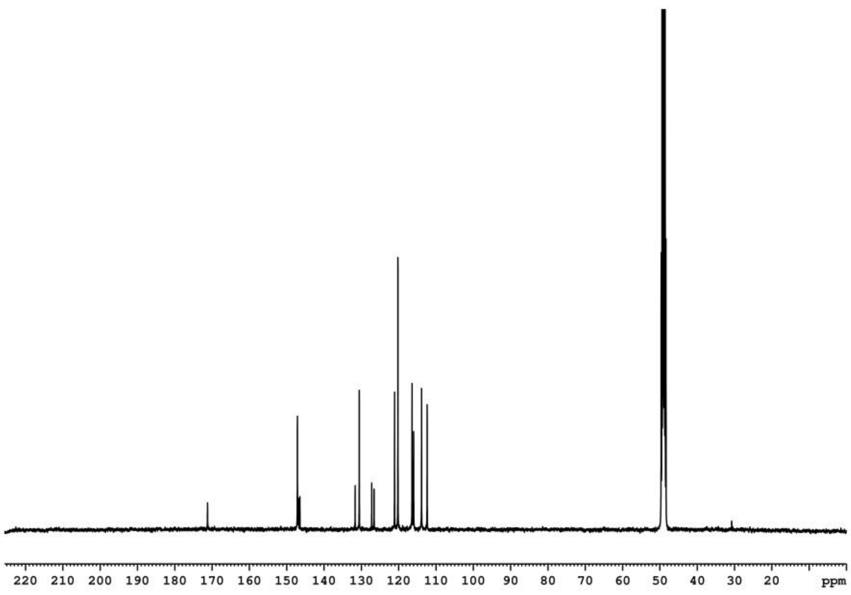

Figure S29. 100 MHz ^{13}C-NMR spectrum of compound **4** in DMSO-d_6.

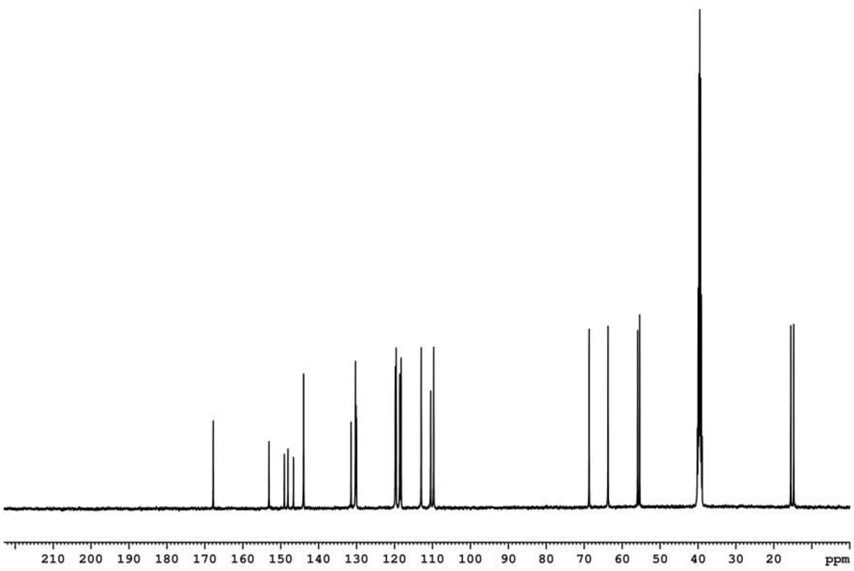

Figure S30. 100 MHz ^{13}C-NMR spectrum of compound **5** in DMSO-d_6.

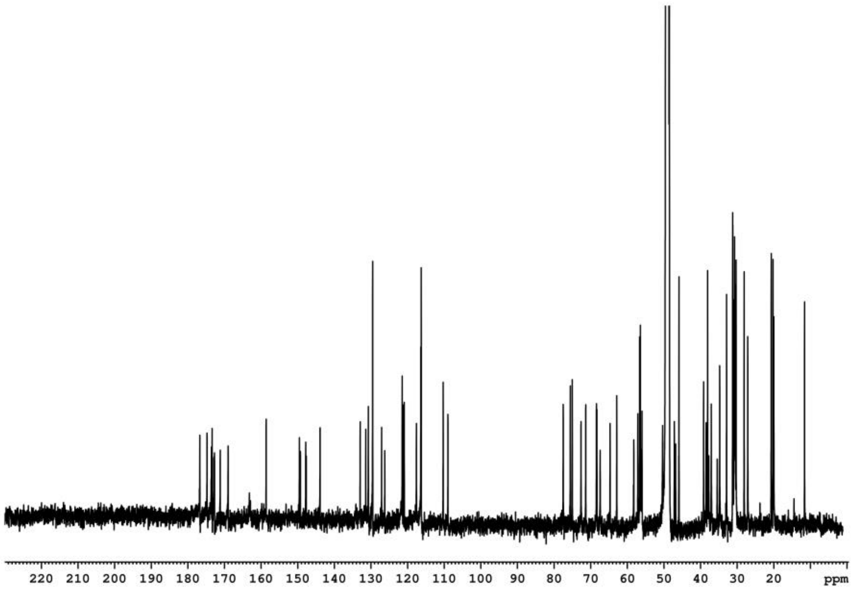

Figure S31. 125 MHz ^{13}C-NMR spectrum of compound **6** in CD_3OD.

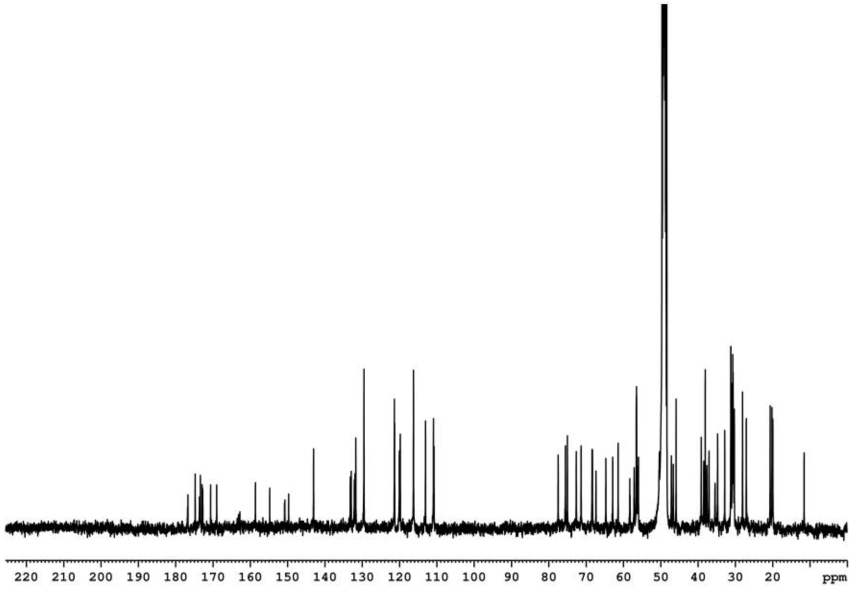

Figure S32. 100 MHz ^{13}C-NMR spectrum of compound **7** in CD_3OD.

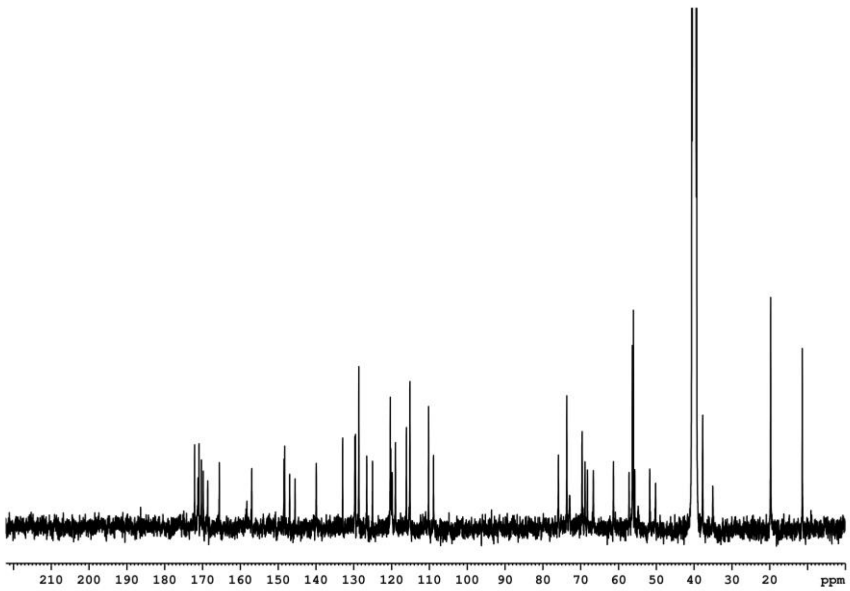

Figure S33. 100 MHz ^{13}C-NMR spectrum of compound **8** in DMSO-d_6.

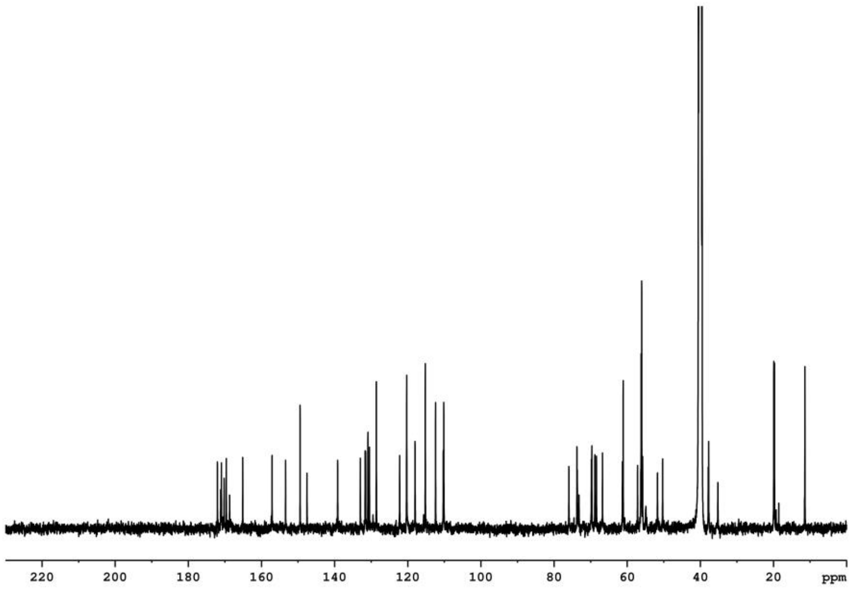

Figure S34. 125 MHz ^{13}C-NMR spectrum of compound **9** in DMSO-d_6.

3. References

(1) Jones, T.; Federspiel, N. A.; Chibana, H.; Dungan, J.; Kalman, S.; Magee, B. B.; Newport, G.; Thorstenson, Y. R.; Agabian, N.; Magee, P. T.; Davis, R. W.; Scherer, S. The Diploid Genome Sequence of Candida Albicans. *Proc. Natl. Acad. Sci. U. S. A.* **2004**, *101* (19), 7329–7334. DOI :10.1073/pnas.0401648101.

(2) Vincent, B. M.; Lancaster, A. K.; Scherz-Shouval, R.; Whitesell, L.; Lindquist, S. Fitness Trade-Offs Restrict the Evolution of Resistance to Amphotericin B. *Public Libr. Sci. Biol.* **2013**, *11* (10). DOI : 10.1371/journal.pbio.1001692.

(3) Chen, Y. L.; Montedonico, A. E.; Kauffman, S.; Dunlap, J. R.; Menn, F. M.; Reynolds, T. B. Phosphatidylserine Synthase and Phosphatidylserine Decarboxylase Are Essential for Cell Wall Integrity and Virulence in Candida Albicans. *Mol. Microbiol.* **2010**, *75* (5), 1112–1132. DOI: 10.1111/j.1365-2958.2009.07018.x.

(4) Brachmann, C. B.; Davies, A.; Cost, G. J.; Caputo, E.; Li, J.; Hieter, P.; Boeke, J. D. Designer Deletion Strains Derived from Saccharomyces Cerevisiae S288C: A Useful Set of Strains and Plasmids for PCR-Mediated Gene Disruption and Other Applications. *Yeast* **1998**, *14* (2), 115–132. DOI: 10.1002/(SICI)1097-0061(19980130)14:2<115::AID-YEA204>3.0.CO;2-2.
